# Hybrid multi-objective optimization of µ-synthesis robust controller for frequency regulation in isolated microgrids

**DOI:** 10.1038/s41598-025-85910-6

**Published:** 2025-01-17

**Authors:** Abdallah Mohammed, Ahmed Kadry, Maged Abo-Adma, Adel El Samahy, Rasha Elazab

**Affiliations:** Faculty of Engineering, Helwan University, Cairo, Egypt

**Keywords:** Optimal µ-synthesis controller, Isolated microgrid, Frequency regulation, Robust performance, Robust stability, Multi-objective optimization, Energy science and technology, Engineering

## Abstract

Frequency regulation in isolated microgrids is challenging due to system uncertainties and varying load demands. This study presents an optimal µ-synthesis robust control strategy that regulates microgrid frequency while enhancing system performance and stability—a proposed fixed-structure approach for selecting performance and robustness weights, informed by subsystem frequency analysis. The controller is optimized using multi-objective particle swarm optimization (MOPSO) and multi-objective genetic algorithm (MOGA) under inequality constraints, employing a Pareto front to identify optimal solutions. Comparative analyses demonstrate that the MOPSO-optimized controller achieves superior robustness and performance, tolerating up to 236% uncertainty compared to 171% for conventional µ-synthesis controllers. Additionally, it significantly reduces frequency deviation and enhances transient response. Nyquist stability analysis confirms robustness across renewable energy uncertainties. The results highlight the proposed controller’s effectiveness in isolated microgrid frequency regulation, with future work focused on discrete-time implementation for practical digital signal processing (DSP) applications.

## Introduction

Isolated microgrids are pivotal for providing reliable and sustainable power to remote and off-grid regions. By utilizing DERs such as solar PV, diesel generators, wind turbines, and ESS, these systems offer improved energy reliability and reduced reliance on fossil fuels. However, their limited inertia, coupled with load fluctuations and uncertainties in renewable energy generation, poses critical challenges for frequency regulation^1,2^. These limitations necessitate the development of advanced control strategies to maintain system stability and performance, ensuring the sustainable and efficient operation of isolated microgrids.

Decentralized control schemes for frequency regulation have been proposed to improve microgrid efficiency and reliability, addressing some of the complexities inherent in isolated systems^3^. Researchers have focused on developing advanced frequency control algorithms^4^. For instance, a centralized MPC algorithm is proposed in^5^ to control isolated microgrid frequency, but it primarily handles load disturbances and overlooks uncertainties in the system. Moreover, centralized control systems are prone to single points of failure, raising reliability concerns. Similarly^6^, presented a centralized control strategy incorporating load uncertainties, but its effectiveness diminishes when faced with high levels of DER uncertainty. These studies, while contributing to frequency regulation, suffer from certain limitations. Most existing traditional control methods, such as those in^5,6^, either fail to address uncertainties in DERs adequately or are significantly impacted by them, resulting in decreased controller performance and stability^7,8^. To effectively address these uncertainties, robust control approaches are essential. However, most robust control techniques face significant challenges using arbitrary performance and robustness weights, which may not optimally reflect the dynamics of individual subsystems in the microgrid^9,10^. Also, robust control techniques employ complex state-feedback controllers with an order equal to or higher than the plant they control. This can lead to implementation and performance degradation difficulties, particularly in high-uncertainty scenarios^9,10^. Several studies have explored robust control approaches for frequency regulation in microgrids. A centralized H ∞ controller was applied in^11,12^, effectively addressing system uncertainties through robust frequency control techniques. Additionally, a centralized H2/H ∞ controller was proposed in^13,14^ to regulate the frequency of interconnected microgrids. Furthermore, a decentralized H ∞ controller was employed in^15,16^, ensuring reliable and secure operation for frequency regulation in isolated microgrids. Fuzzy logic control techniques are widely applied to regulate microgrid frequency. For instance, in^17^, a robust grade-2 fuzzy cascaded controller is introduced for frequency control in AC microgrids. In^18^, a robust type-2 fuzzy cascaded PI fractional-order technique is proposed to ensure frequency stability in PV/Wind systems. Similarly, in^19^, a fractional-order fuzzy power system stabilizer is employed to control the frequency of isolated microgrids. In^20^, a grasshopper-optimized fractional-order multi-stage controller is designed for microgrid frequency regulation. Furthermore, in^21^, fractional inertia control and fractional gradient descent control are proposed for adaptive frequency control of microgrids. A decentralized µ-synthesis robust controller was proposed in^22^ to regulate the frequency of interconnected microgrids. In^23^, a conventional second-order µ-synthesis robust controller is designed for microgrid frequency regulation; however, the performance and robustness weights are determined using a trial-and-error method to ensure each system operates within a specified frequency range. A centralized µ-synthesis robust controller was presented in^24^ to regulate the frequency of isolated microgrids. In^25^, another centralized µ-synthesis robust controller is introduced to address uncertainty parameters and regulate microgrid frequency. In general, the previous robust control studies suffer from two prevalent issues. First, a fixed structure for selecting performance and robustness weights is lacking. Often, these weights are assigned arbitrarily without conducting a comprehensive frequency analysis of each subsystem within the microgrid or determining the appropriate operational bandwidth based on mechanical characteristics. This issue can detrimentally affect the controller’s robust performance and stability. The second issue is that the controller’s order is greater than or equal to the plant order. This results in a complex control structure. Consequently, some research efforts focus on reducing the controller’s order using order reduction techniques^26,27^, such as Hankel optimal model order reduction, as applied in^12^, where the controller order was reduced from 7th to 4th. However, simplifying a controller can lead to losing critical dynamic characteristics, resulting in poorer performance. The reduced-order controller may struggle to achieve the same level of accuracy or responsiveness as the original system^28^.

Research gap and objective of this paper are summarized in two key points. First, the paper addresses the challenges of robust control by introducing a fixed-structure approach for selecting performance and robustness weights based on the frequency analysis of each microgrid subsystem. This method enhances robust performance and stability while ensuring the controller remains low-order, specifically second-order. Second, the proposed controller effectively handles system uncertainties, thereby overcoming the shortcomings of traditional control techniques.

**Fig. 1:**
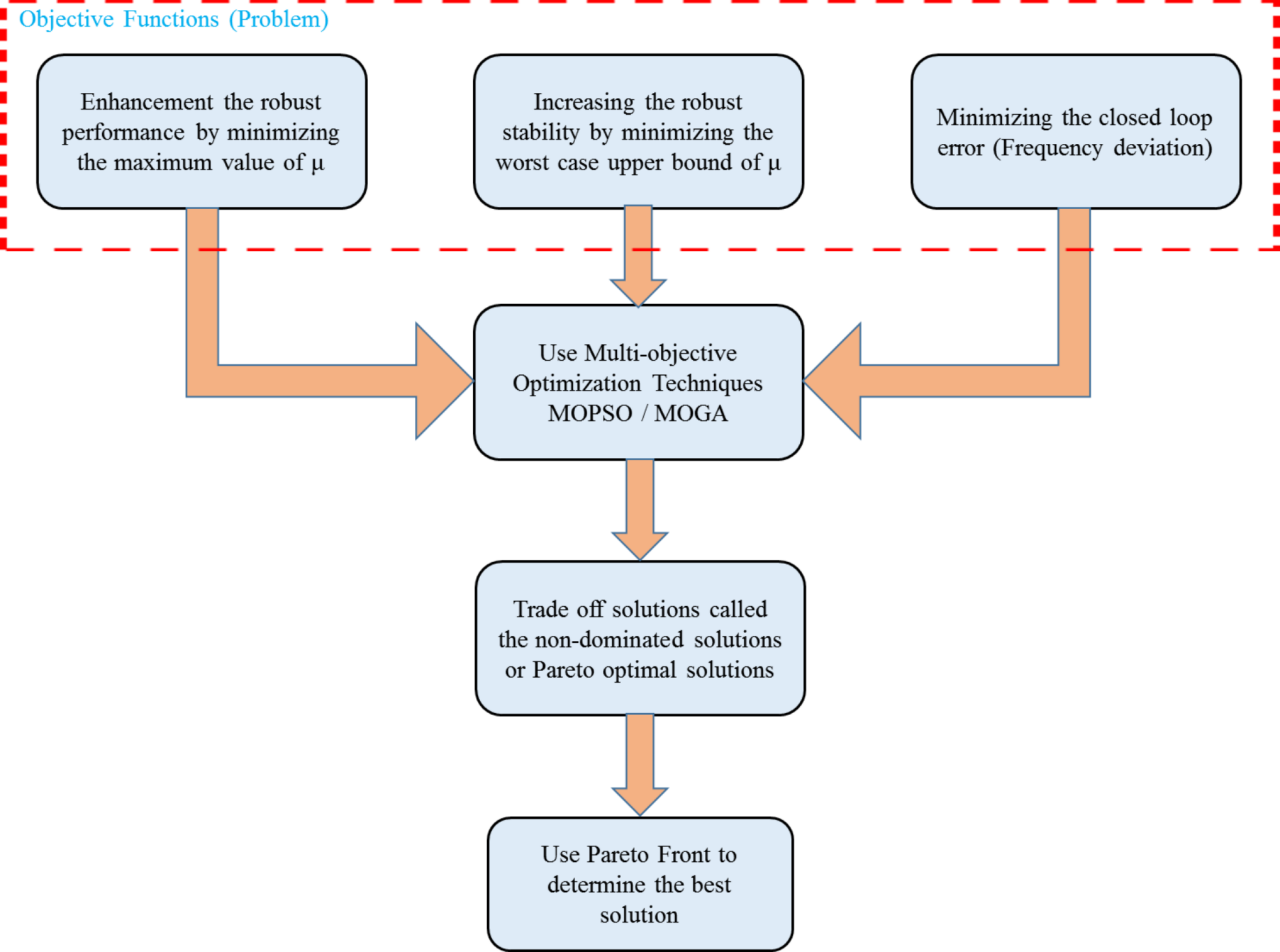
Optimization process for optimal µ-synthesis robust controller.

The key contributions of this study are outlined as follows:

- Structured Weight Selection: A fixed structure is utilized to select performance and robustness weights based on a thorough frequency analysis of microgrid subsystems. This ensures optimal weight assignment, improving robust performance and stability.
- Low-Order Controller Design: A second-order optimal µ-synthesis robust controller is characterized by simplicity and ease of implementation while providing effective frequency regulation in the isolated microgrids.
- Improved Uncertainty Handling: The proposed optimal µ-synthesis robust controller addresses system uncertainties using the coprime factor uncertainty method, which offers superior handling of uncertainties in both the plant and controller compared to other techniques^29^.
- Optimization-Based Controller Design: An optimal µ-synthesis robust controller is proposed and designed using MOGA and MOPSO individually. This approach employs inequality constraints to simultaneously optimize three objective functions: enhancing the robust performance, improving the robust stability, and minimizing system closed-loop error (frequency deviation).
- Hybrid Multi-Objective Optimization: Fig. 1 illustrates the optimization process for the proposed optimal µ-synthesis controller. The MOPSO and MOGA are tested to balance trade-offs among the three objective functions. A Pareto front approach is employed to identify the optimal solution from the non-dominated solutions generated by MOPSO and MOGA. This approach effectively captures the differences between conflicting objectives, enabling a comprehensive evaluation of the solution space and selection of the most balanced design configuration.

The rest of the paper is structured as follows: “Microgrid modeling” section covers the microgrid modeling. “Uncertainty representation” section discusses the representation of uncertainty using coprime factor models. “Optimal design of μ-synthesis controller” section details the design of the proposed optimal µ-synthesis controller, including the multi-objective optimization process for determining the controller parameters. “Simulation results” section presents the simulation results and a comprehensive comparison to the conventional µ-synthesis robust controller. Lastly, “Conclusions” section concludes the paper, highlighting the key contributions and providing insights for future research.

## Microgrid modeling

**Fig. 2:**
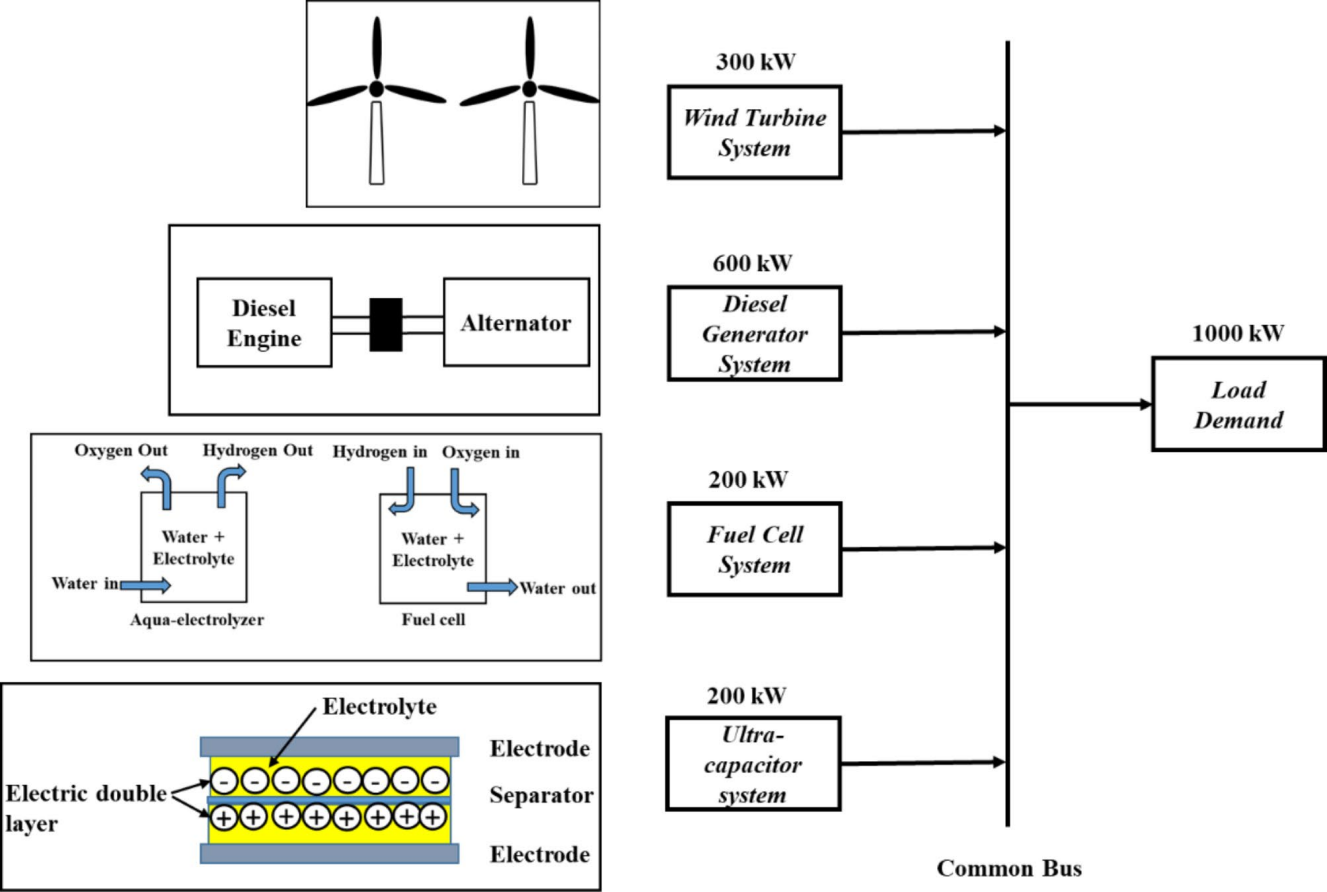
The studied microgrid configurations.

Isolated hybrid microgrids are designed to provide dependable and eco-friendly energy solutions in remote or off-grid areas by integrating multiple renewable energy resources such as wind, solar, and storage systems^30^. The study of frequency control in these microgrids often uses low-order dynamic models, given their low operational frequencies (50–60 Hz) and the typically slow response of subsystems^31^. Simplified dynamic models for microgrids are discussed in detail in several studies^32,33^. The studied microgrid configuration is shown in Fig. 2. It includes a 300 kW wind turbine system, 600 kW diesel generator system, 200 kW fuel cell system, and 200 kW ultra-capacitor system for energy storage, coordinated to meet the microgrid 1000 kW load demand^34^.

Equation (1) illustrates the microgrid power balance concerning the subsystems. Equation (2) depicts the relation between microgrid power and frequency. In accordance with Eq. (2), if variations in frequency arise due to changes in wind speed or fluctuations in load power, subsystems equipped with frequency regulation controllers will adjust their active power output to offset the discrepancy between generation and load^35^.

1 $$\:{\varDelta\:P}_{L}=\:{\varDelta\:P}_{D}+{\varDelta\:P}_{F}+{\varDelta\:P}_{WG}-{\varDelta\:P}_{A}-{\varDelta\:P}_{U}$$

2 $$\:(Ms+D)\varDelta\:F\left(S\right)=\:{\varDelta\:P}_{D}\left(s\right)+{\varDelta\:P}_{F}\left(s\right)+{\varDelta\:P}_{WG}\left(s\right)-{\varDelta\:P}_{A}\left(s\right)-{\varDelta\:P}_{U}\left(s\right)-{\varDelta\:P}_{L}\left(s\right)$$

**Fig. 3:**
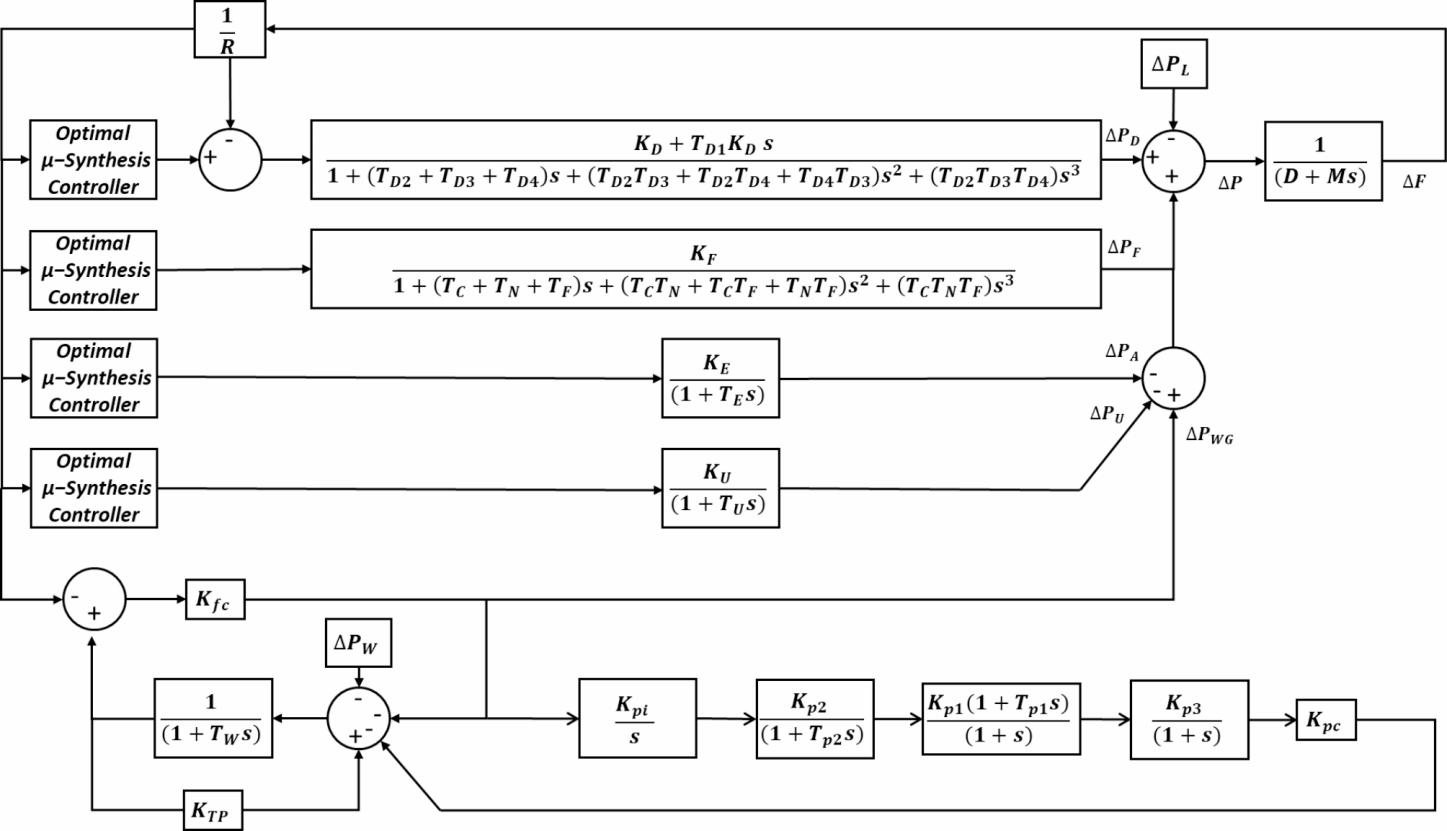
The proposed system transfer function.

Where $\:M,\:D,\:and\:\varDelta\:F$ denote the inertia constant, damping coefficient, and the change in microgrid frequency, respectively. The challenge involves coordinating the operation of each generation unit within specific frequency ranges, taking into consideration the distinct dynamics inherent to each unit, as explained in the following sections. Figure 3 illustrates the transfer function block diagram of the proposed system. Table 1 shows the proposed microgrid parameters^34^.

**Table 1:** Microgrid parameters^34^.

Parameter	Value	Parameter	Value
$\:{K}_{F}$	0.01	$\:{K}_{E}$	0.04
$\:{K}_{C}$	1	$\:{K}_{U}$	-0.7
$\:{K}_{N}$	1	$\:{\text{T}}_{\text{E}}$	0.002
$\:{T}_{C}$	0.004	$\:{\text{T}}_{\text{U}}$	0.9
$\:{T}_{N}$	0.04	R	5
$\:{T}_{F}$	4	$\:{\text{K}}_{\text{D}}$	0.4
M	0.4	$\:{\text{T}}_{\text{p}1}$	0.6
D	0.03	$\:{\text{T}}_{\text{p}2}$	0.04
$\:{\text{K}}_{\text{f}\text{c}}$	1.5	$\:{\text{K}}_{\text{p}\text{c}}$	0.08

## Uncertainty representation

Uncertainty refers to the discrepancies or errors between mathematical models and real-world systems, and the method used to capture these deviations is known as an uncertainty representation. These representations differ based on the level of structure they incorporate and are broadly categorized into structure and unstructured uncertainties^36^. Uncertainty in plant models can arise from several factors^37,38^: (1) parameters in the linear model are often only approximately known or can vary due to changes in the operating conditions, (2) imperfections in measurement devices, (3) at high frequencies, the structure and order of the model may be unknown, with uncertainties potentially exceeding 100%, and (4) even with a detailed model, one may choose a simplified nominal model and treat the neglected dynamics as uncertainties.

### Structure uncertainty modeling

In this case, the model’s structure is well-defined, but certain parameters remain uncertain. Parametric uncertainty is typically characterized by assuming the uncertain parameter has a specific bounded range [$\:{\alpha\:}_{min},$ $\:{\alpha\:}_{max}$]^36^. Where $\:{\alpha\:}_{P}$ is expressed by Eqs. (3), (4), and (5).

3 $$\:{\alpha\:}_{P}=\:\stackrel{-}{\alpha\:}\:(1+{r}_{d}\varDelta\:$$

4 $$\:\stackrel{-}{\alpha\:}\:=\:\frac{\varDelta\:\:({\alpha\:}_{min}+{\alpha\:}_{max})}{2}$$

5 $$\:{r}_{d}\:=\:\frac{\varDelta\:\:\left({{\alpha\:}_{max}-\:\alpha\:}_{min}\right)/2}{\stackrel{-}{\alpha\:}}$$

While Δ is a scalar value such that $\:\left|{\Delta\:}\right|$ ≤1.

### Unstructured uncertainty modeling

In this type, the model exhibits errors due to missing dynamics, typically at higher frequencies, either because of intentional simplifications or a lack of knowledge about the physical process. Every real-world system model will inherently possess this type of uncertainty. Unstructured uncertainty is commonly represented in three forms: adaptive uncertainty, multiplicative uncertainty, and coprime factor uncertainty. Equations (6), (7), and (8) present the mathematical models for adaptive, multiplicative, and coprime factor uncertainties, respectively^29^.

6 $$\:{P}_{\varDelta\:}\left(s\right)=P\left(s\right)+\:{W}_{1}\varDelta\:{W}_{2}\:$$

7 $$\:{P}_{\varDelta\:}\left(s\right)=\left(1+{W}_{1}\varDelta\:{W}_{2}\right)\:P\left(s\right)$$

8 $$\:{P}_{\varDelta\:}\left(s\right)={\left(M+{\varDelta\:}_{M}\right)}^{-1}\:\left(N+{\varDelta\:}_{N}\right)$$

Where $\:{W}_{1},{\:W}_{2},\:M,\:N$ represent the transfer functions that model the plant’s unstructured uncertainty.

**Fig. 4:**
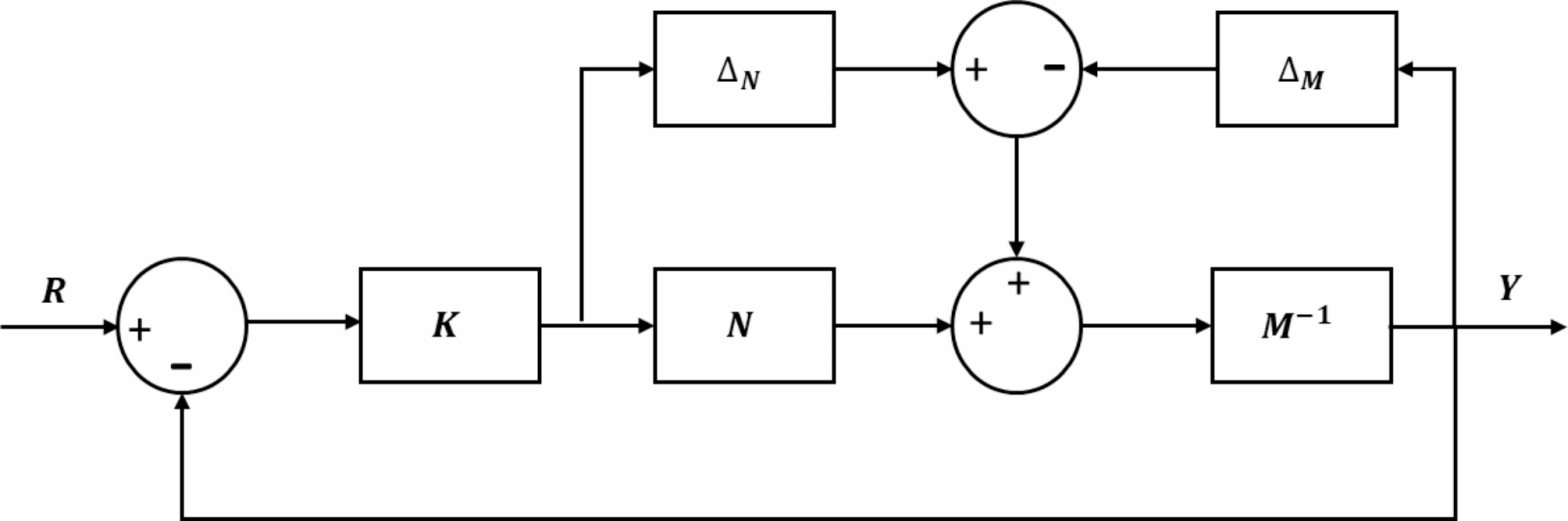
Block diagram of the plant with coprime factor uncertainty.

Many modern studies focus on system uncertainty to ensure stability and optimize performance in real-time simulations. For instance^39^, introduces an adaptive sliding mode control approach with adaptive gains, fine-tuned using stochastic gradient descent, to address uncertainty issues. Similarly^40^, proposes a PID controller integrated with stochastic gradient descent to tackle the uncertainty challenge. Moreover^41^, employs an extended grey wolf optimizer to overcome the limitations of traditional population-based algorithms in effectively managing uncertainty and nonlinear system dynamics. This paper presents an optimal µ-synthesis robust controller designed to address system uncertainties using coprime factor uncertainty representation. This method showcases significant advantages in control system design and analysis, particularly in ensuring robustness. By explicitly incorporating uncertainties in the plant parameters, it facilitates the development of control systems that are inherently robust to disturbances and parameter variations^29^. The approach addresses uncertainties in both the numerator and denominator of the plant transfer function, offering a comprehensive framework for robust control design. This makes it highly effective for real-time simulations, where capturing dynamic behaviors and maintaining stability under real-world operating conditions are essential. Figure 4 illustrates the block diagram of the plant with coprime factor uncertainty, demonstrating its practical application in modeling and mitigating uncertainties.

## Optimal design of µ-synthesis controller

The µ-synthesis theorem provides a framework for designing controllers that minimize the worst-case structured singular value (µ) while adhering to specific constraints. These controllers are tailored to ensure robust stability and high performance, even in the presence of structured and unstructured uncertainties. The design process involves iterative optimization to determine the controller parameters that achieve the desired robustness and performance objectives. The following subsections delve into the fundamental aspects of µ-synthesis, including its principles, weight selection strategies, and optimization methods.

### Basic of µ-synthesis controller

**Fig. 5:**
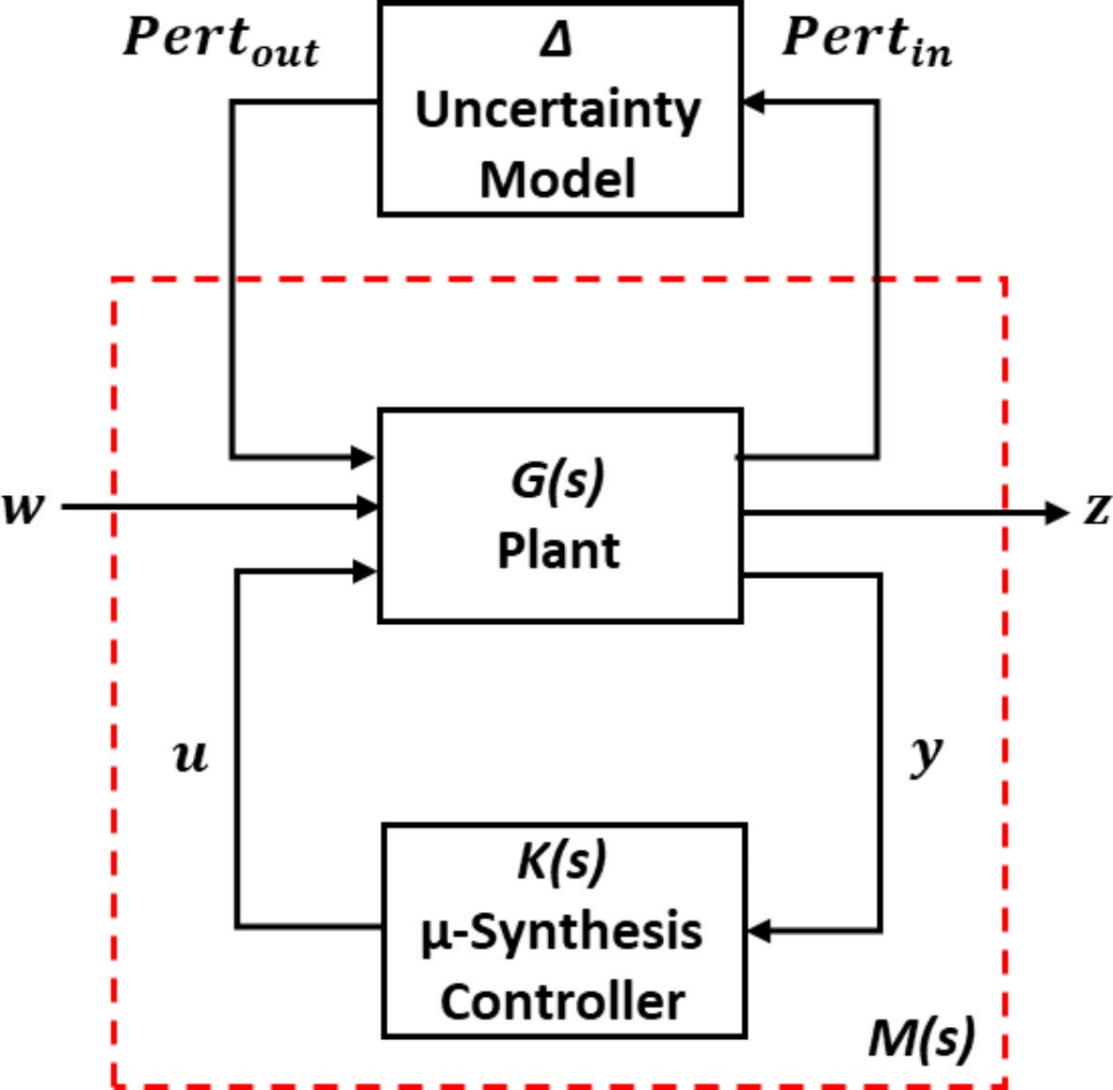
The standard configuration for M-Δ for µ-synthesis robust controller.

The standard configuration for M-Δ for µ-synthesis controller is depicted in Fig. 5. In this diagram, $\:\:{Pert}_{in}$, $\:{Pert}_{out}$, *u*,* w*, *y*, and *z* are input perturbations, output perturbations, control signals, exogenous inputs, measured outputs, and control performance signals, respectively^42^.

The µ-synthesis controllers are implemented in the feedback control loop to regulate the behavior of the system while considering uncertainties. The design of the controller depends on the system’s model, the specified performance requirements, and the characteristics of the structured and unstructured uncertainties^43^.

The D-K iteration technique is employed in order to iteratively refine the µ-synthesis controller design to achieve robust performance and robust stability with respect to uncertainties in the system. The process involves alternately updating the controller parameters and the weighting functions used in the µ-analysis until convergence is achieved^44^.

Robust stability and performance should be considered in the design process of µ-synthesis controller. Robust stability ensures that the system remains stable under all conditions of uncertainty. Robust performance, on the other hand, requires that the output error gain stays below a specified threshold across different frequencies despite variations in system parameters. The robust performance and stability criteria are determined in Eqs. (9) and (10), respectively^45^.

9 $$\:{\mu\:}_{max}\:<1\:$$

10 $$\:{\mu\:}_{ub}\:<1\:$$

### Performance and robustness weight selection strategy

**Fig. 6:**
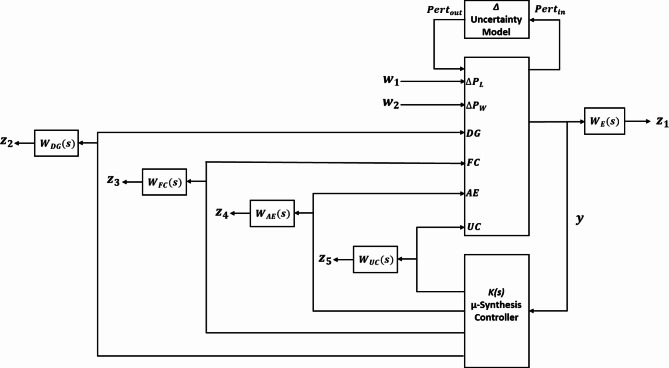
Closed-loop configuration for the proposed system.

Performance weights are applied to control actions to minimize control sensitivity. Robustness weights ensure that each generation unit operates within its specified frequency range, aligned with its mechanical structure. The performance weight (error weight) function is denoted by $\:{W}_{E}\left(s\right)$ in Fig. 6, while the robustness weight functions (subsystem weights) are represented by ($\:{W}_{GG}\left(s\right),\:{W}_{FC}\left(s\right),\:{W}_{AE}\left(s\right),\:and\:{W}_{UC}\left(s\right)$).

The performance weights are selected to minimize the cost function in Eq. (11), where *W*_*lp*_ is the sensitivity function low pass filter and *W*_*hp*_ is the complementary sensitivity high pass filter. The weighting functions of S can be obtained by Eq. (12)^29,42^. Noise is not considered in this work; therefore, *W*_*hp*_ is set to zero^46–48^.

11 $$\left\| {\left[ {\begin{array}{*{20}l} {S*W_{{lp}} } \\ {T*~W_{{hp}} } \\ \end{array} } \right]} \right\|_{2}$$

12 $$W_{{lp}} \left( s \right) = \frac{{~{s \mathord{\left/ {\vphantom {s {M_{P} }}} \right. \kern-\nulldelimiterspace} {M_{P} }} + ~w_{C} }}{{s + \varepsilon *w_{C} }}$$

**Fig. 7:**
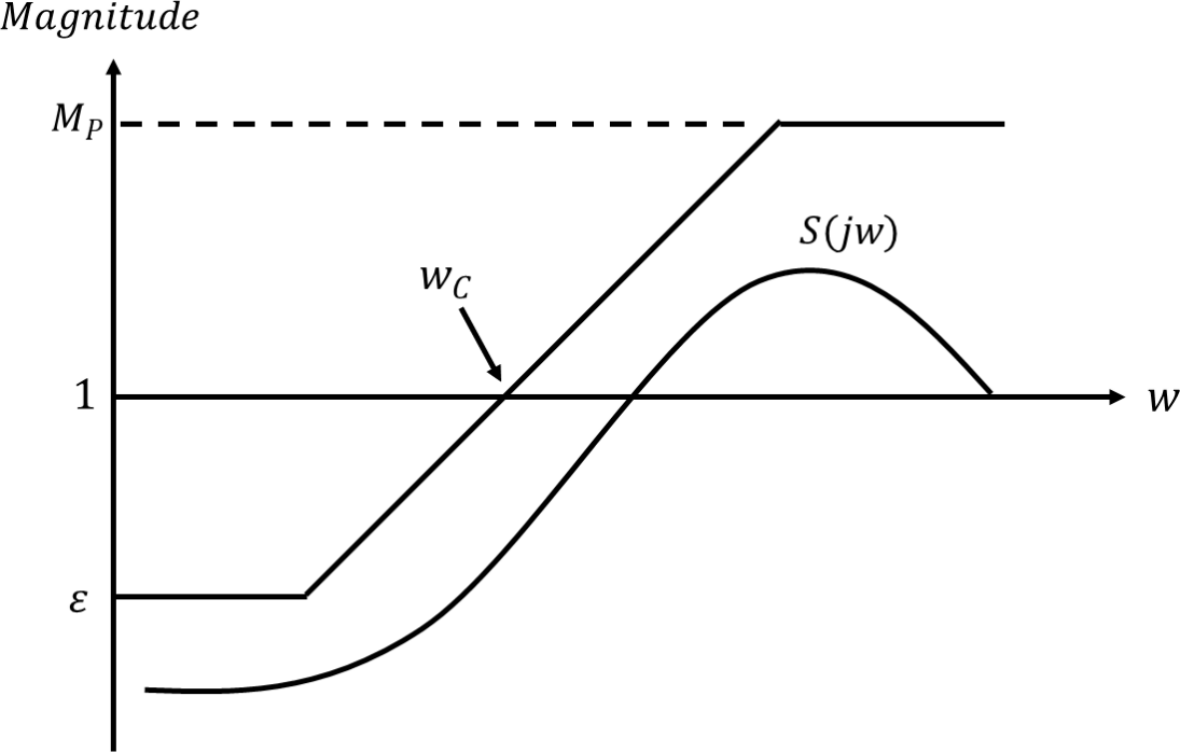
Weight specifications for sensitivity function.

Figure 7 illustrates the process for determining the weight specifications for the sensitivity function, including $\:\:{w}_{C}$, $\:{M}_{P}$, and $\:\epsilon\:$.

**Fig. 8:**
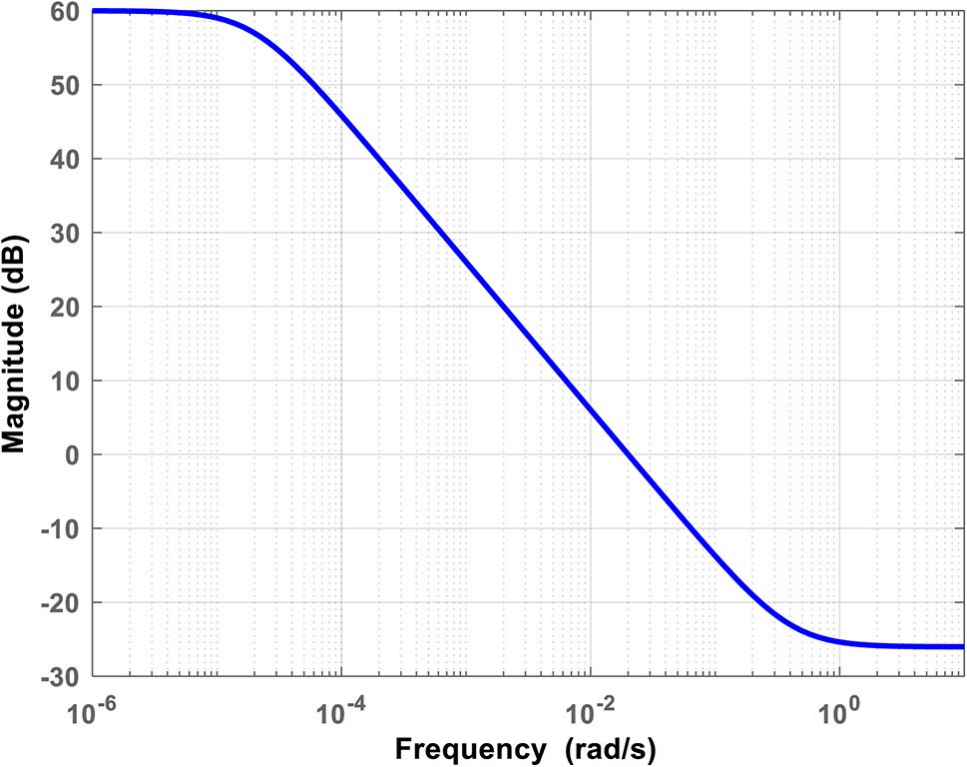
Performance weight bode diagram.

The Bode diagram for the performance weight $\:{W}_{E}\left(s\right)$ is shown in Fig. 8, based on Eq. (11) and the desired specifications presented in Fig. 7.

The robustness weight functions ($\:{W}_{GG}\left(s\right),\:{W}_{FC}\left(s\right),\:{W}_{AE}\left(s\right),\:and\:{W}_{UC}\left(s\right)$) as shown in Fig. 6, are selected to ensure each unit operates within a specific frequency band. For example, the ultra-capacitor operates in the high-frequency band. In contrast the diesel generator, fuel cell, and aqua-electrolyzer operate in the low-frequency band due to their mechanical characteristics. The general form of the low-pass and high-pass filters is determined in Eq. (13)^29,42^. Where $\:a,b,\:and\:{K}_{w}$ are constants, the conditions of low-pass and high-pass filters are $\:(a\:>b)$ and ($\:b\:>a$), respectively.

13 $$\:W\left(S\right)=\:{K}_{w}\frac{\:s+\:a}{s+b}$$

**Fig. 9:**
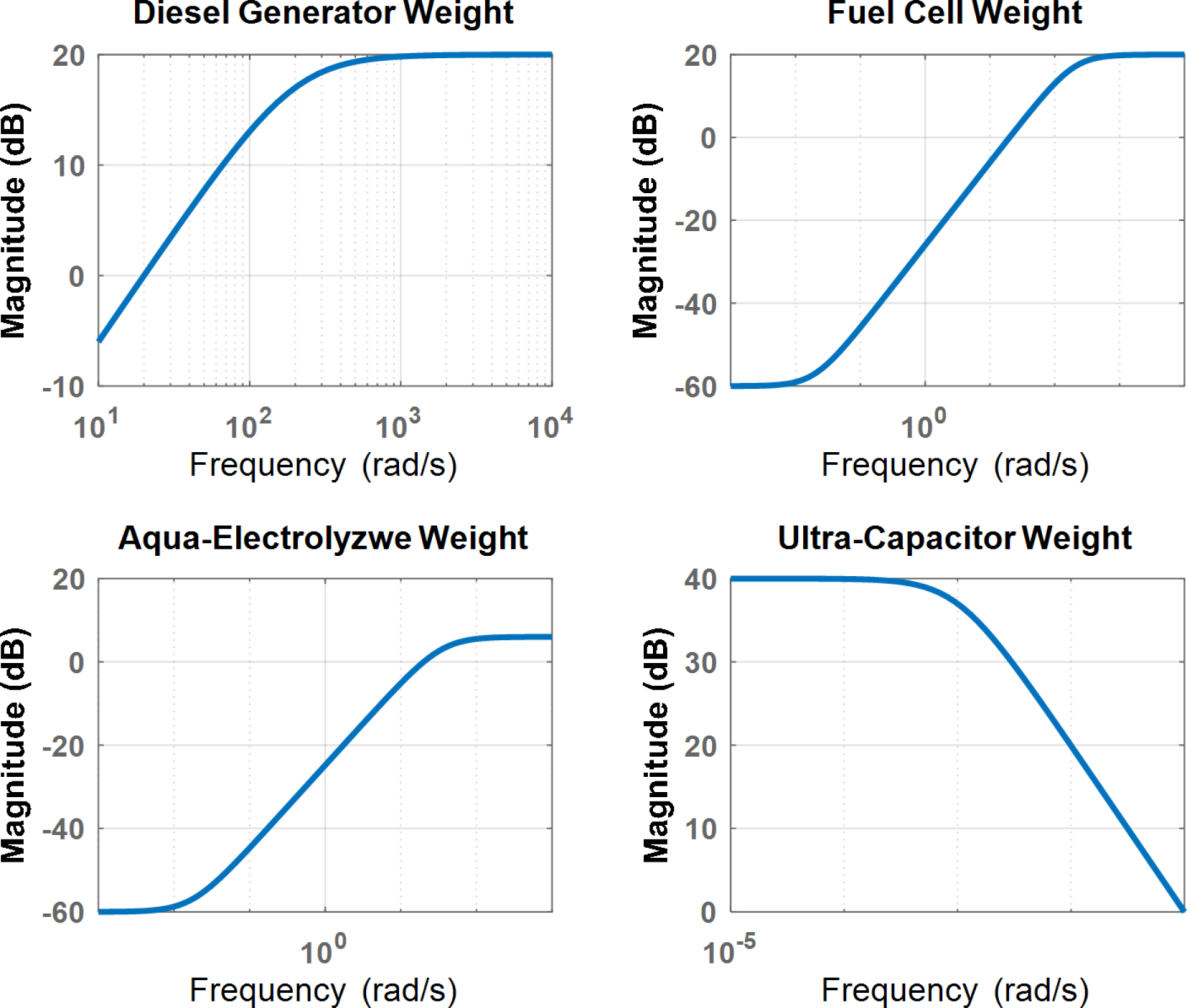
Robustness weight bode diagram.

Figure 9 shows the Bode diagrams for the robustness weight functions. $\:{W}_{UC}\left(s\right)$ is a low-pass filter designed to operate the ultra-capacitor in the high-frequency band. Similarly, $\:{W}_{GG}\left(s\right),\:{W}_{FC}\left(s\right),\:{W}_{AE}\left(s\right)$ are designed as high-pass filters to operate the aqua-electrolyzer, diesel generator, and fuel cell in the low-frequency band.

**Fig. 10:**
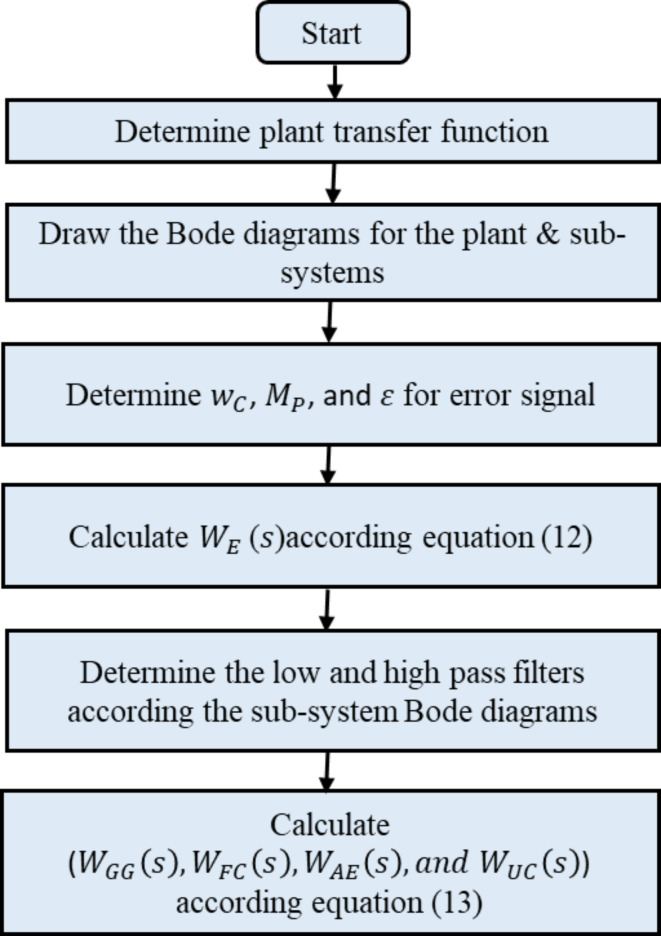
Performance and robustness weights selection flowchart.

The initial performance and robustness weights are determined following the flowchart shown in Fig. 10. The first step involves identifying the transfer function of the entire microgrid system and capturing its dynamic behavior. In the second step, Bode diagrams for the entire plant and each sub-system are drawn to represent their frequency responses. In the third step, the performance weight parameters ($\:{w}_{C}$, $\:{M}_{P}$, and $\:\epsilon\:$) are determined, and the performance weight ($\:{W}_{E}\left(s\right)$) is calculated using Eq. (12).

The fourth step involves analyzing the Bode diagrams of each subsystem to design appropriate low-pass and high-pass filters, ensuring optimal operation within designated frequency bands. Finally, the robustness weights ($\:{W}_{GG}\left(s\right),\:{W}_{FC}\left(s\right),\:{W}_{AE}\left(s\right),\:and\:{W}_{UC}\left(s\right)$) for each subsystem are calculated using Eq. (13) to enforce the desired frequency bands and enhance the control system’s robustness against uncertainties and disturbances.

### Problem definition

This paper presents a multi-objective optimization problem with inequality constraints used to design an optimal µ-synthesis robust controller for the frequency regulation of isolated microgrid proposed in Fig. 3. The controller is characterizes by covering the robust controller challenges by employing a fixed-structure approach for selecting performance and robustness weights based on detailed frequency analysis of each microgrid subsystem, enhancing both robust performance and stability. Additionally, it is designed as a low-order controller, specifically of second-order, ensuring efficiency and practicality. The controller also adeptly manages system uncertainties, surpassing the capabilities of traditional controllers. Three objective functions are considered in the optimization process, as indicated in Eq. (14). The first function ($\:{f}_{1})\:$enhances the robust performance by minimizing the maximum value of µ, the second function ($\:{f}_{2})\:$increases the robust stability by minimizing the worst-case upper bound of µ, and the third function minimizes the closed loop error ($\:{f}_{3})$. The constraints are indicated in Eq. (15).

14 $$\begin{aligned} Minimize~\;~f = & \left[ {~f_{1} ,f_{2} ,f_{3} } \right] \\ f_{1} = & ~\mu _{{max}} \\ f_{2} = & ~\mu _{{ub}} \\ f_{3} = & ~\mathop \smallint \limits_{0}^{\infty } \left| {e\left( t \right)} \right|dt \\ \end{aligned}$$

15 $$\begin{aligned} Inequality~\;constraints\;~a_{{UC}} ~ > & b_{{UC}} \\ a_{{GG}} ~ < & b_{{GG}} \\ a_{{FC}} ~ < & b_{{FC}} \\ a_{{AE}} ~ < & b_{{AE}} \\ \end{aligned}$$

### Optimization techniques

Numerous heuristic techniques have been developed to address multi-objective optimization challenges in the electric power sector, including voltage control, reactive power control, power system operation, power system security, and capacitor placement. The heuristic method proposed in this work employs an iterative generation process that guides subordinate heuristics to identify optimal solutions to the optimization problem efficiently. This study uses MOGA and MOPSO are used to solve the multi-objective optimization problem, subject to the inequality constraints defined in Eqs. (14) and (15). The optimization process involves 12 variables subject to inequality constraints, as defined in Eq. (15). These variables are optimized to develop the desired weight transfer functions, detailed in Subsection 4.2. The control parameters for MOPSO and MOGA are listed in Table 2.

**Table 2:** MOPSO and MOGA control parameters.

Control parameter	Value
Number of populations	100
Number of iterations	300
Number of variables	12 [$\:{K}_{wUC},\:{a}_{UC},\:{b}_{UC},\:{K}_{wGG},\:{a}_{GG},\:{b}_{GG},{K}_{wFC},\:{a}_{FC},\:{b}_{FC},{K}_{wAE},\:{a}_{AE},\:{b}_{AE}]$
Initial values	[0.0001,1000, 0.001,2,0.00995,199,10,0.0199,199,2,0.01732,34.64]
Lower bound	[1e-5,100,1e-4,1e-3,1e-5,10,1e-3,1e-3,10,1e-3,1e-3, 1]
Upper bound	[1,1100,1,50,1,250,50,10,250,50,10,100]

In multi-objective optimization problems, unlike single-objective optimization, a set of non-dominated or Pareto-optimal solutions typically exists (often referred to as the Pareto front). These solutions represent a state where no objective can be improved without degrading at least one objective. When considering all objectives, no other solutions in the search space outperform these Pareto-optimal solutions^49^. Identifying the Pareto front involves two steps, with the first being determining non-dominated solutions if one of the conditions specified by Eq. (16) is met. The second step is determining CD according to Eq. (17). Higher CD means the best solution^50^.

16 $$\:\:\:{f}_{l}\left({x}_{i}\right)\le\:{f}_{l}\left({x}_{j}\right)\:\:\:\:for\:all\:l=\text{0,1},2\dots\:\dots\:.,n$$

$$\:{f}_{l}\left({x}_{i}\right)\le\:{f}_{l}\left({x}_{j}\right)\:\:\:\:for\:at\:least\:one\:l\in\:\{\text{0,1},2\dots\:\dots\:.,n\}$$

17 $$\:\:\:{CD}_{j}^{i}=\frac{{f}_{l}\left({x}_{i+1}\right)\:-{f}_{l}\left({x}_{i-1}\right)}{{f}_{l}^{max}\:-\:{f}_{l}^{min}}$$

**Fig. 11:**
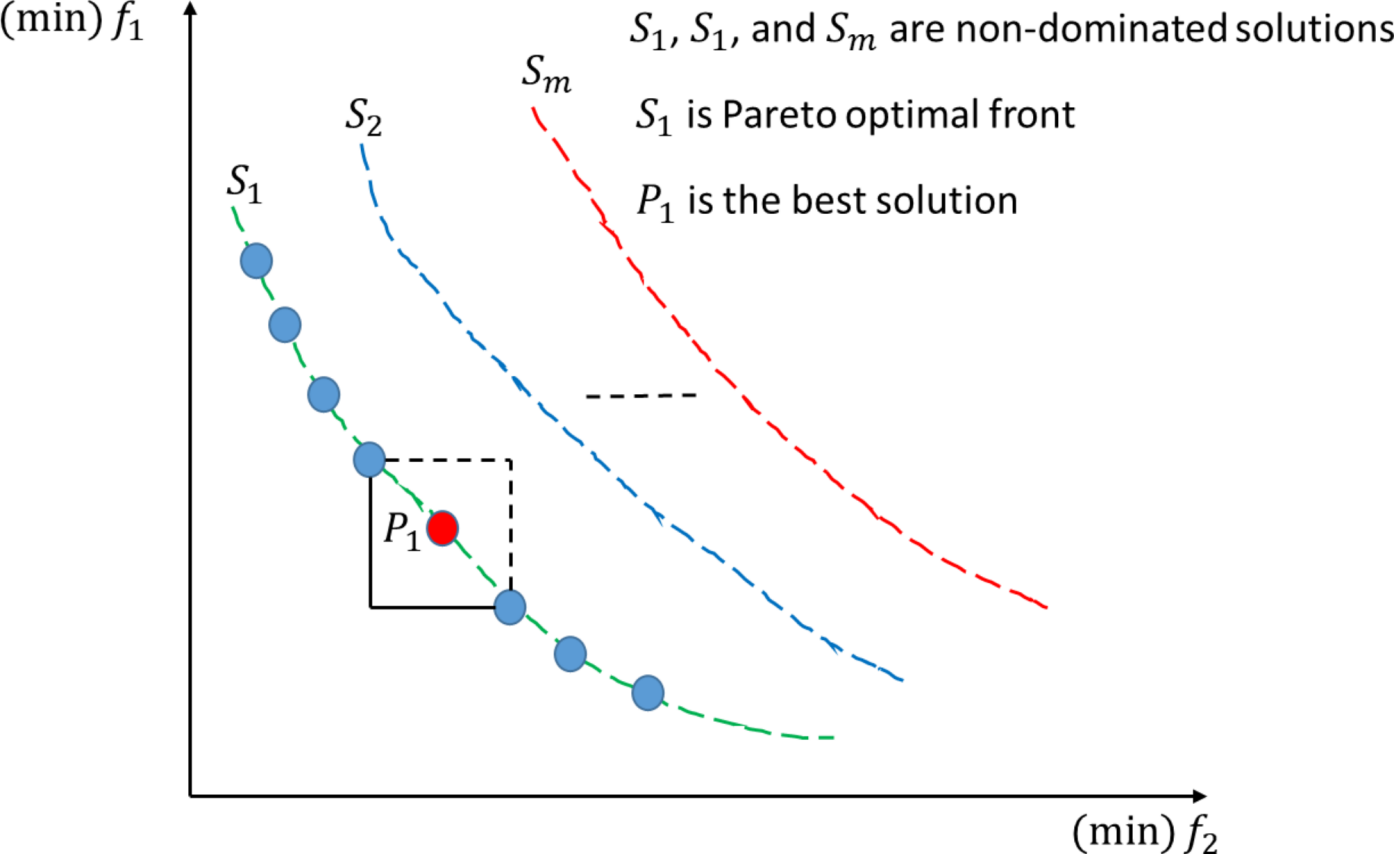
Non-dominated solutions.

Figure 11 shows different non-dominated solutions ($\:{S}_{1},{S}_{2},\:and\:{S}_{m})$ for minimization of $\:{(f}_{1})\:$and $\:{(f}_{2})$. Where $\:{S}_{1}$is the Pareto optimal front and $\:{P}_{1}$is the best solution determined by CD.

**Fig. 12:**
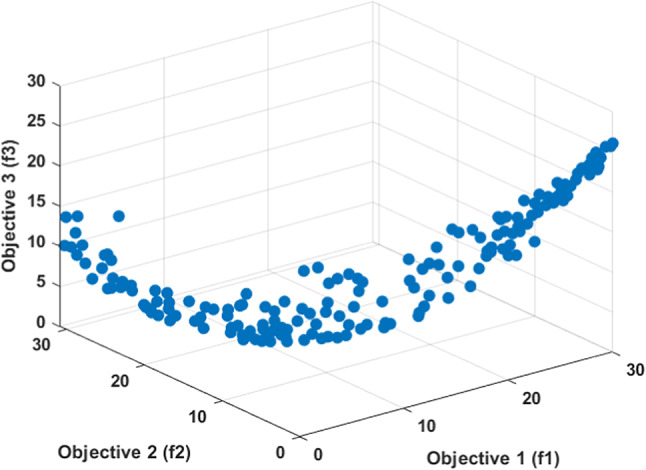
Pareto front for the optimization process.

Figure 12 illustrates the Pareto front for the optimization process, highlighting the three objective functions under consideration.

The design of the optimal µ-synthesis controller involves several technical considerations to ensure system performance and robustness. The following steps outline the key techniques, challenges, and strategies used in determining the µ-synthesis controller parameters:

- Initialization:
  The system dynamics and uncertainty model were defined, and initial performance and robustness weights were selected, as detailed in Sects. 2, 3, and 4. This step ensures a structured starting point for optimization.

- Defining Objectives:
  Objective functions and constraints were formulated based on Eqs. (14) and (15). These objectives balance performance metrics with robustness requirements, such as stability margins and transient response.

- Designing the µ-Synthesis Controller:
  The µ-synthesis controller parameters were designed iteratively using D-K synthesis, as outlined in Eq. (18).
  18 $$\:{C}_{{\upmu\:}}\left(S\right)=\:\frac{{\alpha\:}_{1}*{s}^{2}+{\alpha\:}_{2}*s+{\alpha\:}_{3}}{{\beta\:}_{1}*{s}^{2}+{\beta\:}_{2}*s+{\beta\:}_{3}}\:\:\:\:\:\:\:\:\:\:\:\:\:\:\:\:\:\:\:\:\:\:\:\:\:\:\:\:\:\:\:\:\:\:\:\:\:\:\:\:\:\:\:\:\:\:\:\:\:\:$$
  This process involves iterative refinement of the controller (K) and the scaling matrices (D) to minimize the structured singular value (µ) and improve robustness. Each iteration evaluates objective functions to identify the optimal parameters for the current step.

- Updating Robustness Weights:
  Robustness weights were iteratively updated to balance robustness and performance. The system’s transient and steady-state responses guided adjustments, ensuring convergence to a practical and effective solution.

- Pareto Optimization:
  A Pareto front approach was used to identify optimal controller parameters that satisfy all objectives and constraints. This method ensures a trade-off between conflicting goals, such as performance and robustness, while avoiding over-tuning.

During the optimization process, specific challenges were encountered and addressed, such as:

- Over-Tuning Risks:
  Proper initialization and careful selection of weight filters were critical to avoiding over-tuning, which could lead to overly sensitive controllers. Weight filters were designed to balance performance and robustness without compromising system stability.

- Constraint Management:
  Constraints were carefully defined to ensure the optimization remained within practical bounds.

- Convergence Difficulties:
  The iterative nature of D-K synthesis can sometimes lead to slow convergence. This was mitigated by fine-tuning initial parameters and using the Pareto front to guide the selection of feasible solutions.

**Fig. 13:**
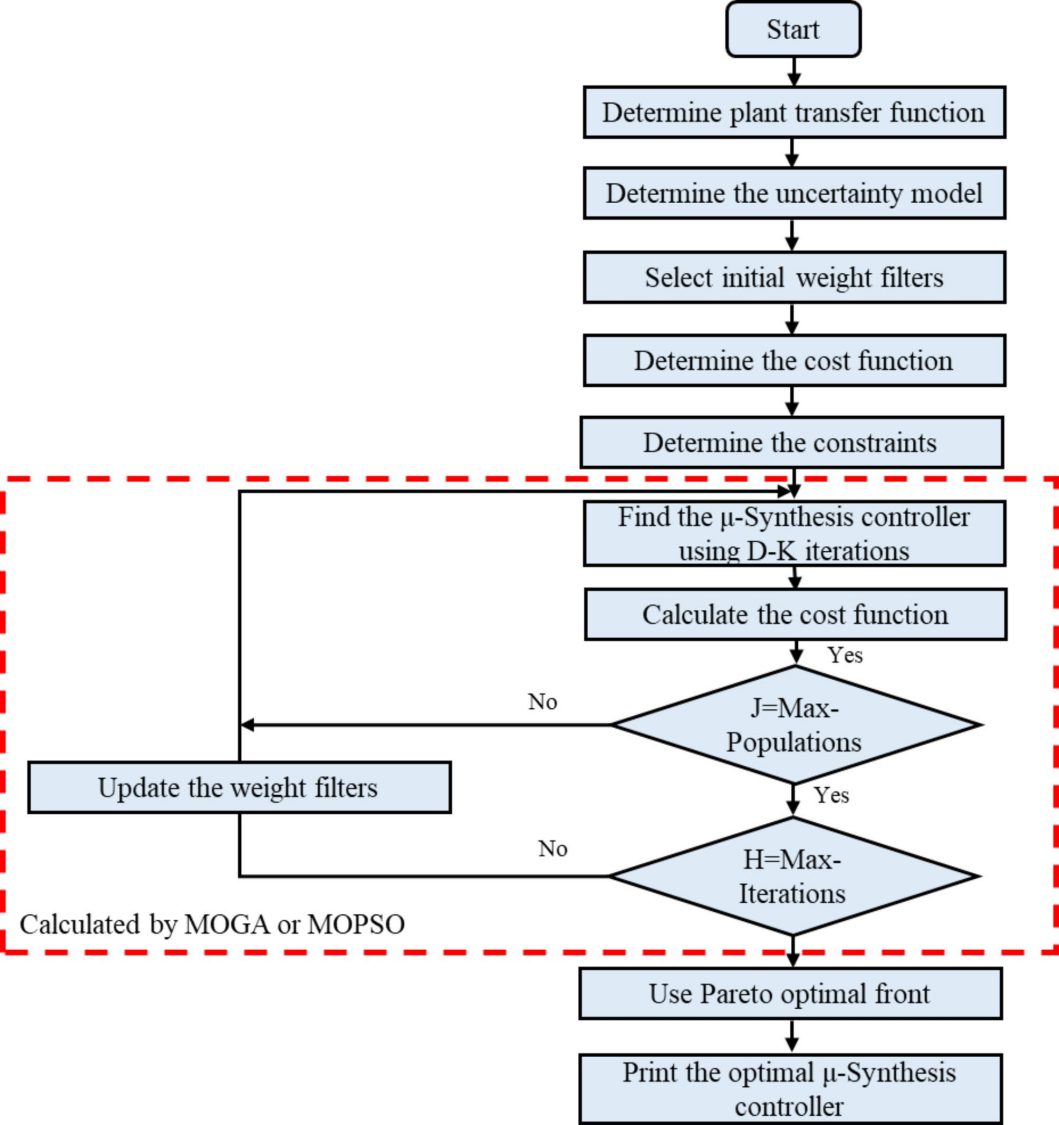
The proposed controller flowchart.

**Fig. 14:**
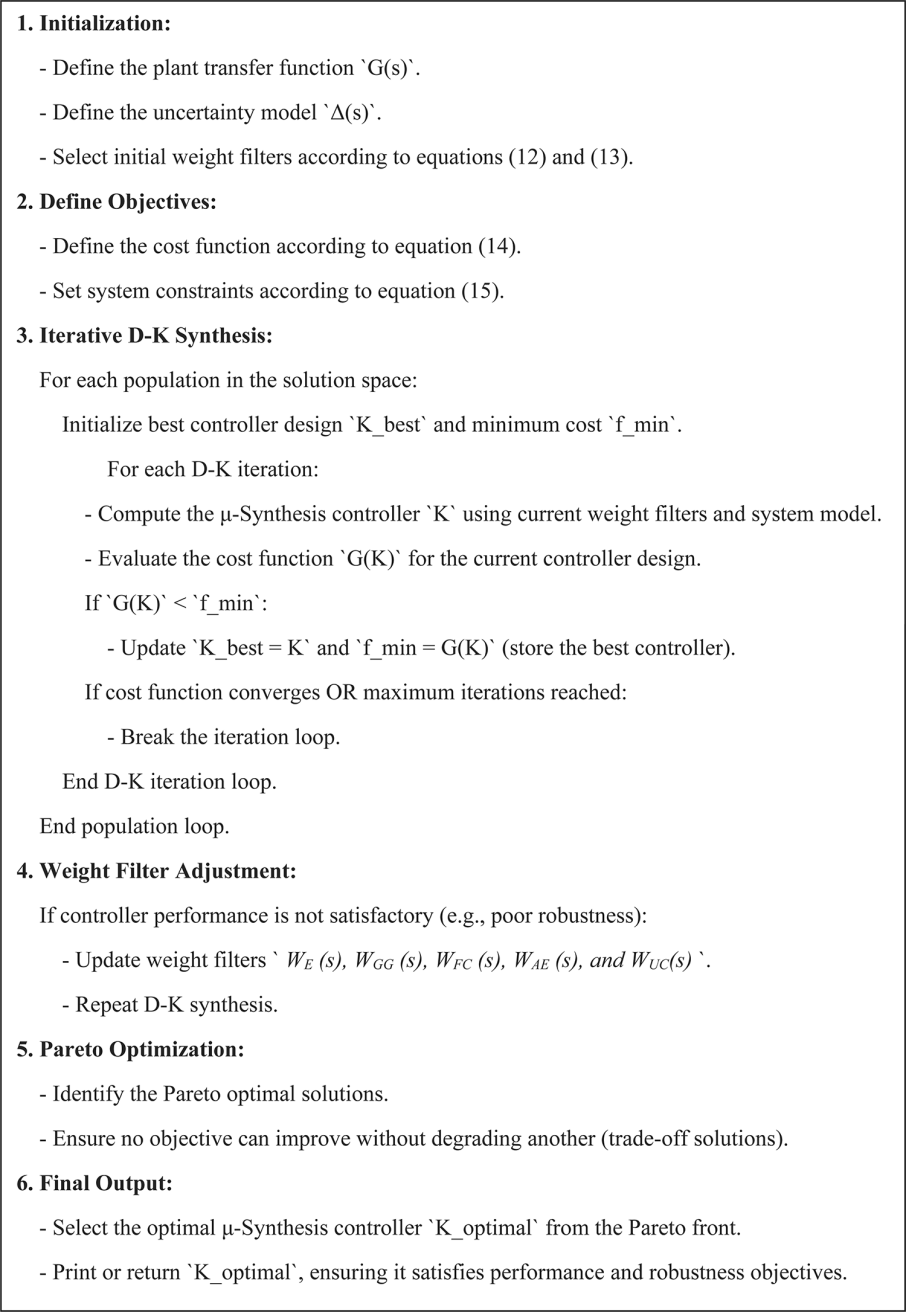
The proposed controller code.

Figures 13 and 14 illustrate the proposed µ-synthesis controller’s design flowchart and the implemented code, providing a detailed visualization of the optimization process.

## Simulation results

In this section, the conventional µ-synthesis controller, as implemented in^23^, is utilized for a comparative study against the proposed optimal µ-synthesis controller. This comparison aims to demonstrate the effectiveness of the proposed optimal controller in maintaining microgrid frequency stability under unstructured uncertainties in system parameters and varying load power conditions. Additionally, the controller’s actions for each subsystem are examined to assess their performance. The comparative analysis emphasizes the superior capability of the optimal µ-synthesis controller in addressing uncertainties and dynamic fluctuations, thereby validating its robustness, performance, and stability in practical applications.

**Fig. 15:**
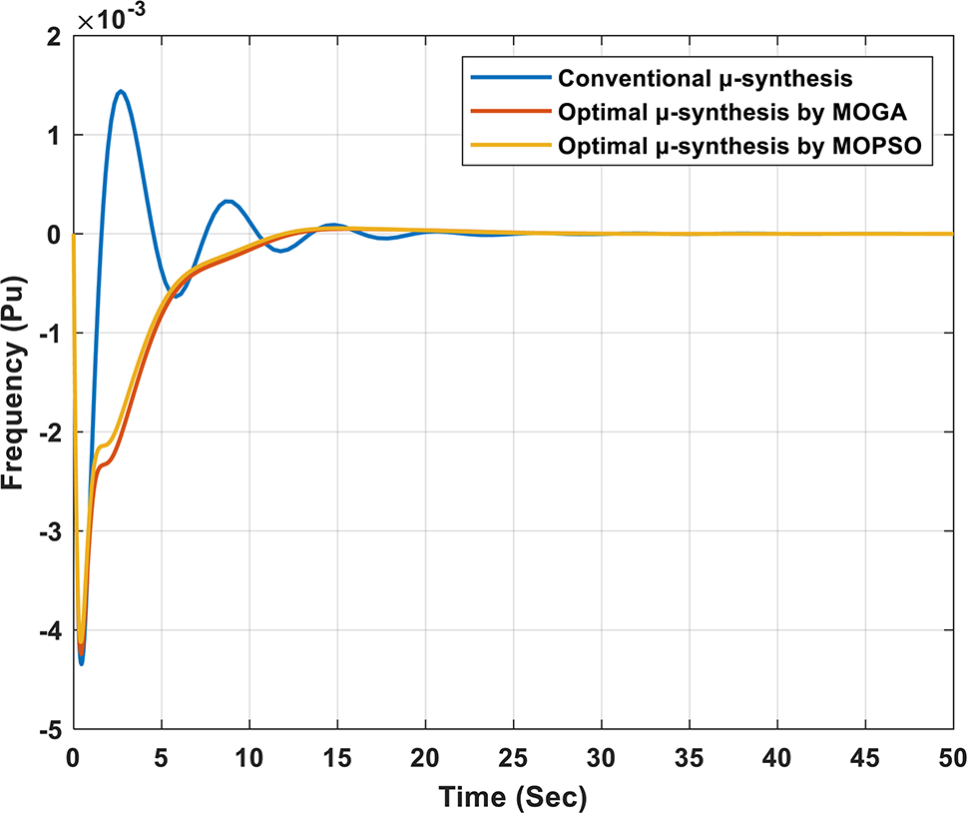
Frequency response of the isolated microgrid under a 5% change in load power.

Figure 15 presents the frequency response of the studied isolated microgrid following a 5% change in load power. The results show that the proposed optimal controller significantly outperforms the conventional controller in managing frequency deviations during transient and steady-state conditions. In particular, the steady-state error is reduced to 7.0491e-9 with the optimal µ-synthesis MOPSO-optimized controller, compared to 2.7935e-8 with the MOGA-optimized controller and 9.0012e-7 with the conventional µ-synthesis controller. These results highlight the proposed controller’s improved transient and steady-state performance, validating its effectiveness in ensuring system stability and robustness under varying operating conditions.

**Fig. 16:**
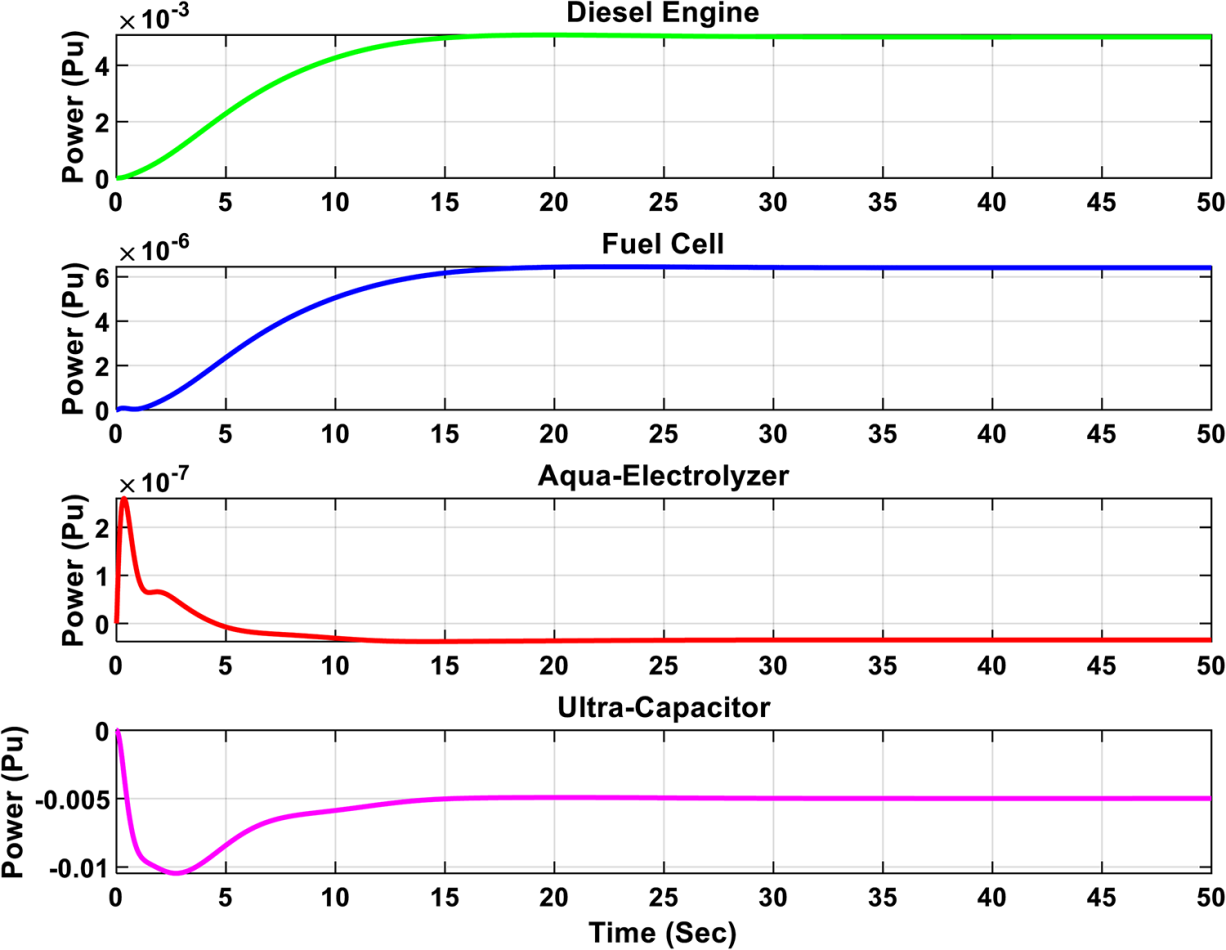
Output power of each subsystem in case of 5% change in load power for the optimal µ-synthesis controllers optimized by MOPSO.

**Fig. 17:**
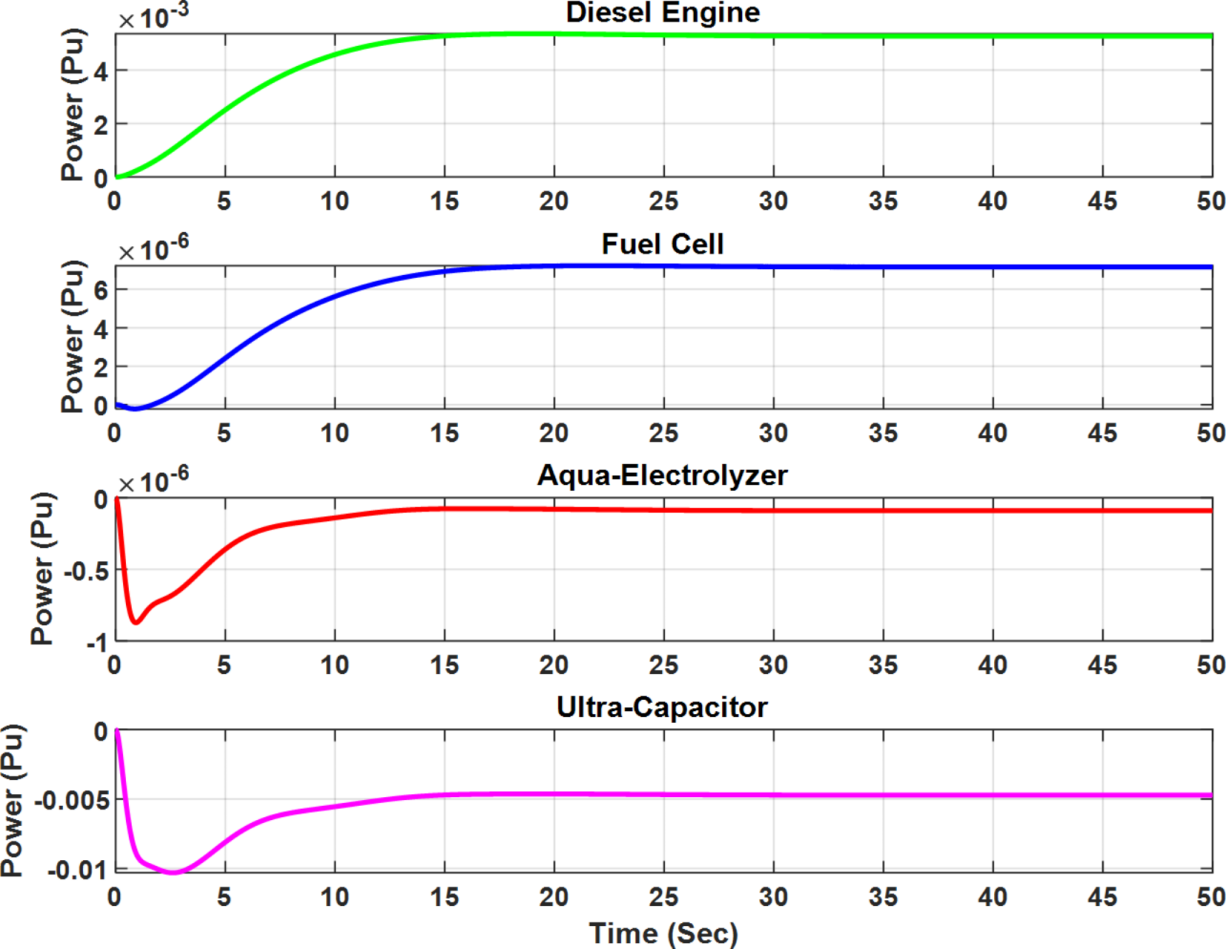
Output power of each subsystem in case of 5% change in load power for the optimal µ-synthesis controllers optimized by MOGA.

Figures 16 and 17 illustrate the output power of each subsystem for the optimal µ-synthesis controllers optimized by MOPSO and MOGA, respectively, in response to a 5% load power change. A detailed analysis shows that ultra-capacitors are effectively utilized within the microgrid, operating in high-frequency bands to meet load demands in these specific frequency ranges. This strategic deployment of ultra-capacitors enhances the system’s dynamic response and plays a crucial role in maintaining overall system stability by compensating for rapid fluctuations in load and generation. These findings demonstrate the advanced control capabilities of the proposed controller configurations.

**Fig. 18:**
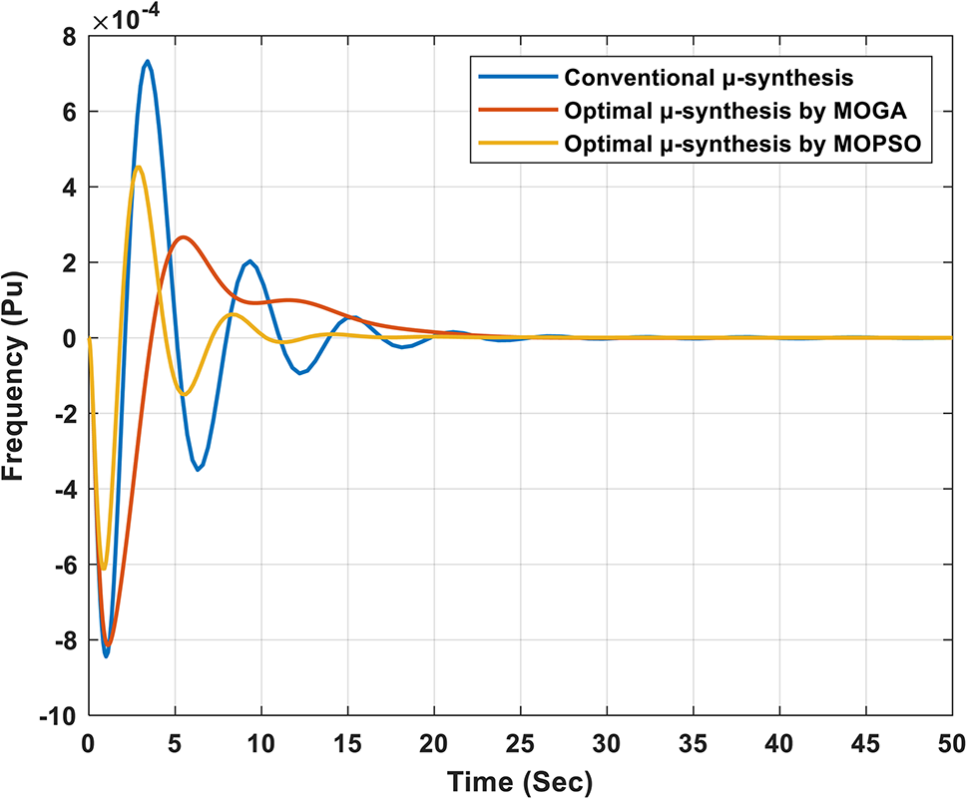
Frequency response of the isolated microgrid under a 5% change in wind power.

**Fig. 19:**
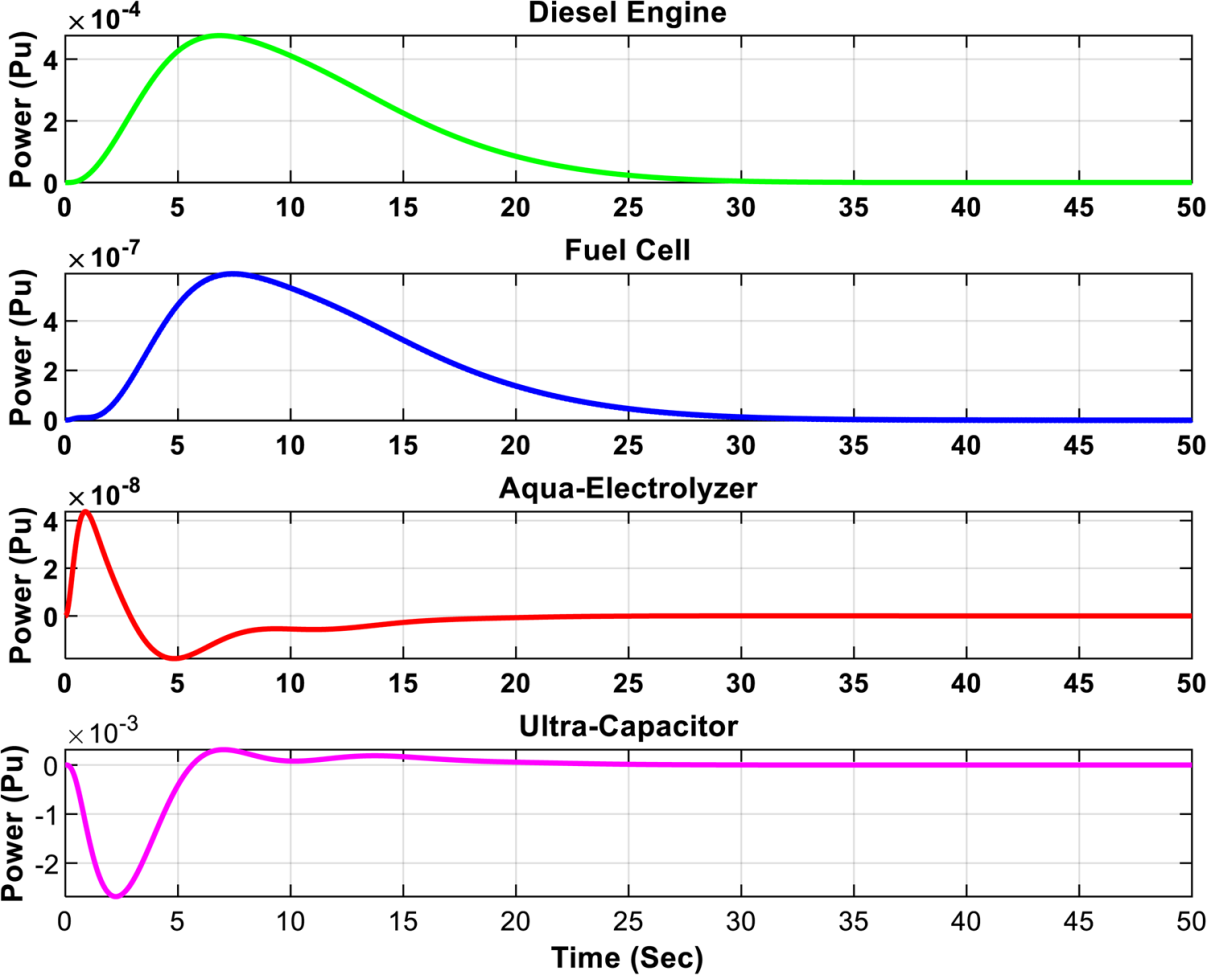
Output power of each subsystem in case of 5% change in wind power for the optimal µ-synthesis controllers optimized by MOPSO.

**Fig. 20:**
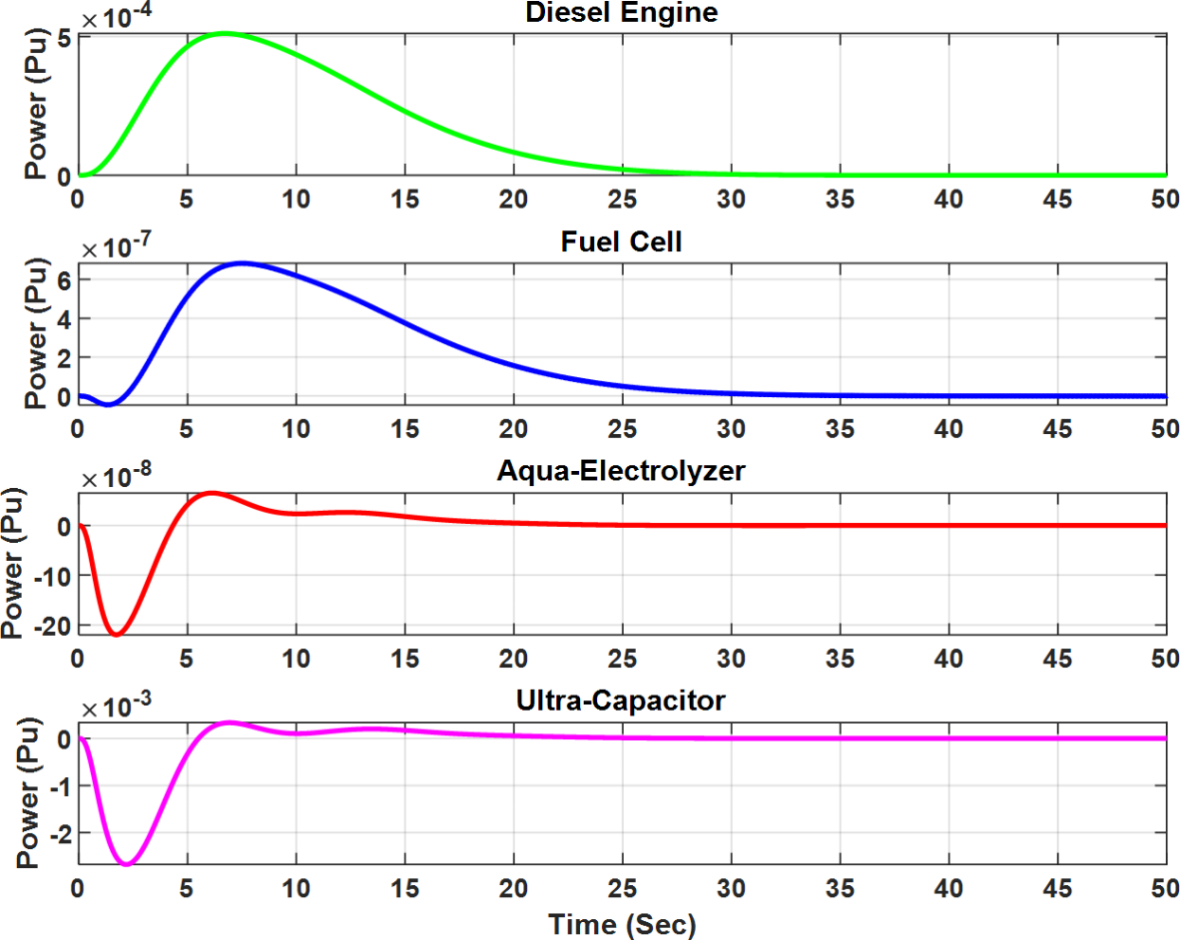
Output power of each subsystem in case of 5% change in wind power for the optimal µ-synthesis controllers optimized by MOGA.

Figure 18 shows the frequency response of the isolated microgrid under a 5% change in wind power. The steady-state error is minimized to 3.2e-9 when using the optimal µ-synthesis MOPSO-optimized controller, compared to 2.8e-8 with the MOGA-optimized controller, and 2.9e-7 with the conventional µ-synthesis controller. Also, Figs. 19 and 20 present the output power of each subsystem in case of 5% change in wind power for the optimal µ-synthesis controllers optimized by MOPSO and MOGA, respectively.

**Fig. 21:**
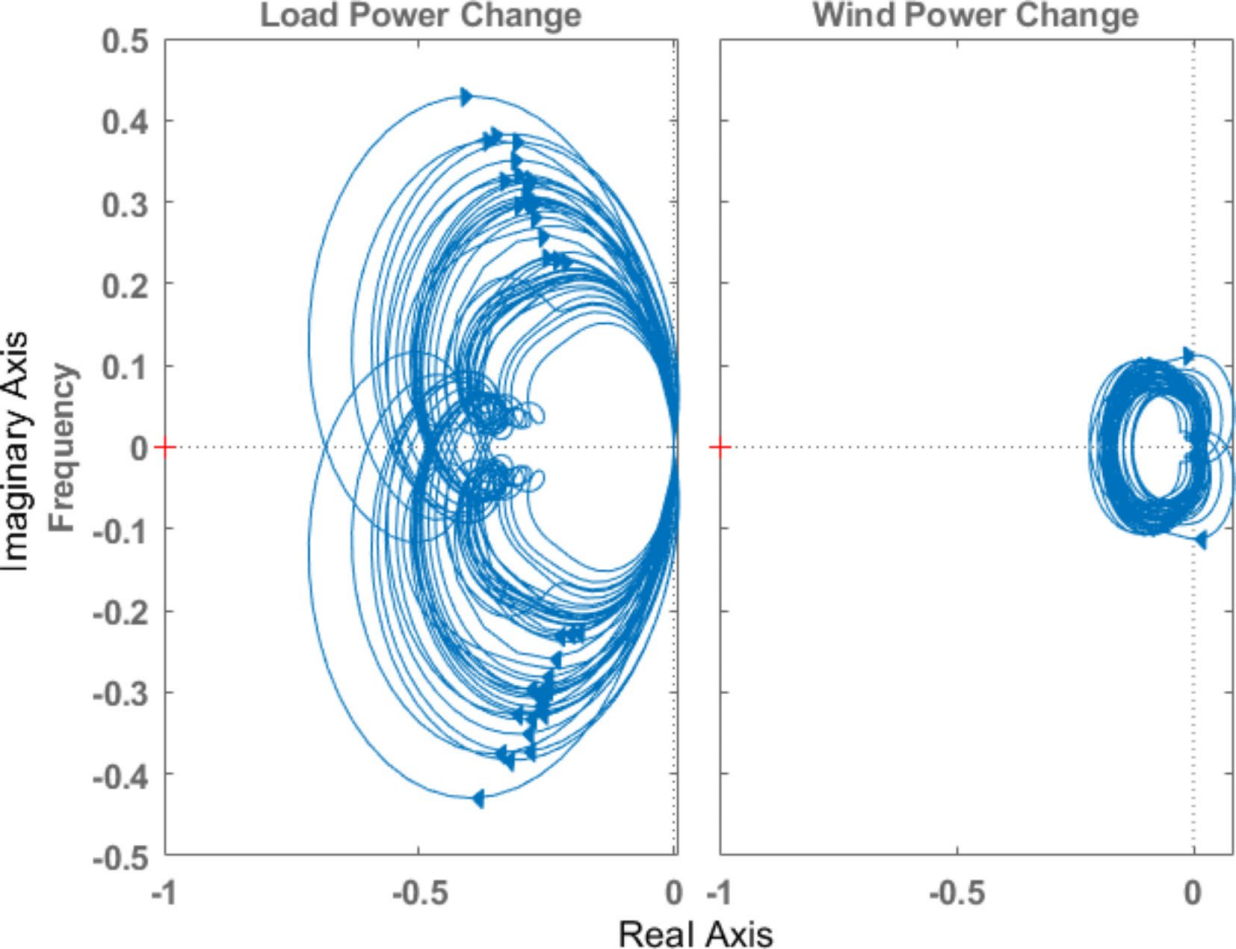
Nyquist diagram for the system in case of optimal µ-synthesis controllers optimized by MOPSO.

**Fig. 22:**
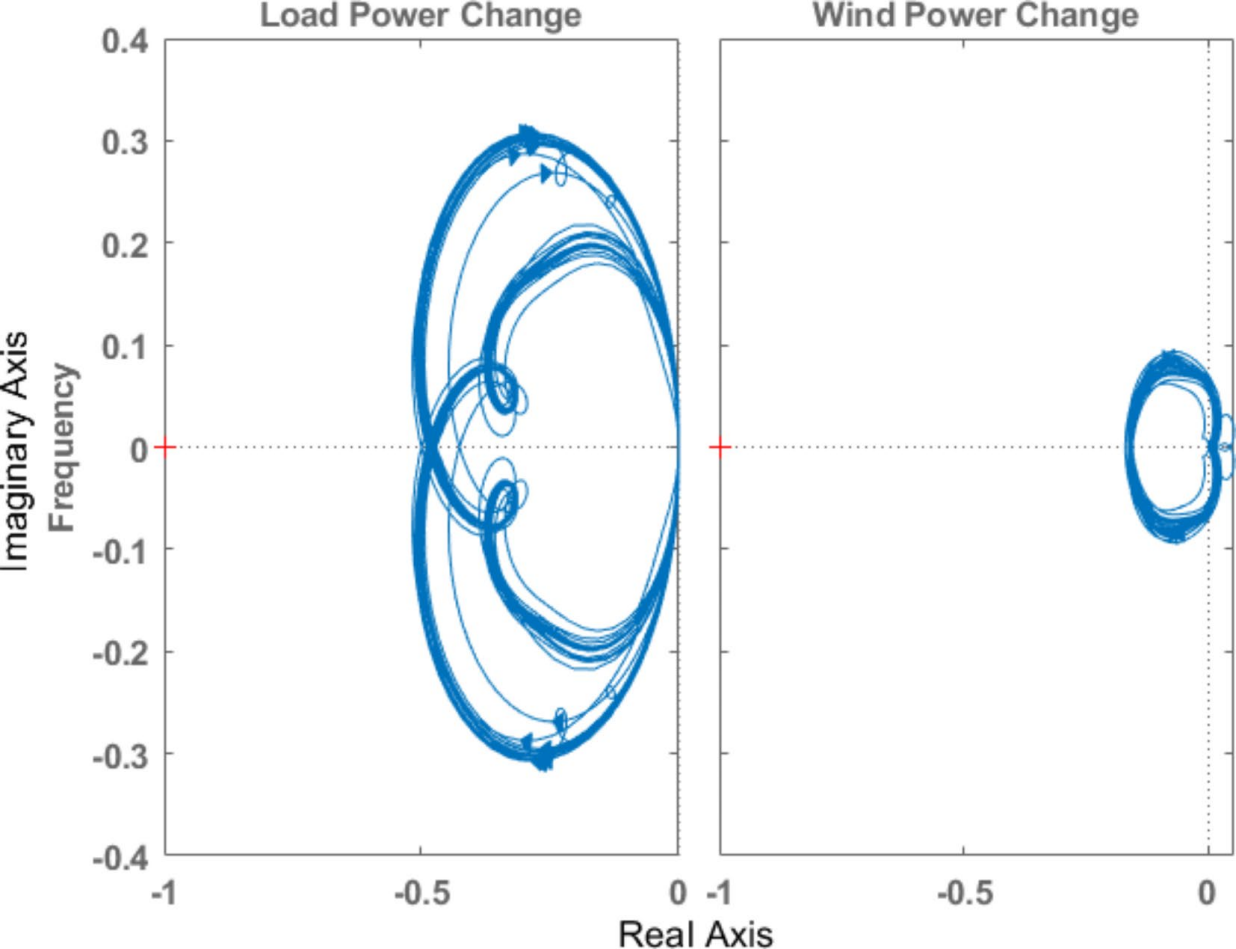
Nyquist diagram for the system in case of optimal µ-synthesis controllers optimized by MOGA.

The stability analysis of the system equipped with optimal µ-synthesis controllers is conducted using the Nyquist Criterion, as illustrated in Figs. 21 and 22 for controllers optimized by MOPSO and MOGA, respectively. According to the Nyquist Criterion, the system maintains stability across a broad spectrum of uncertainties and load disturbances, indicating the robustness of the proposed controllers. The Nyquist plots demonstrate that the closed-loop poles remain within stable regions despite the presence of significant unstructured uncertainties, validating the controllers’ ability to maintain system stability under varying operational conditions. This analysis underscores the effectiveness of the proposed optimal µ-synthesis robust controllers in providing reliable frequency regulation in isolated microgrids.

The steady-state and dynamic characteristics of the proposed controller are compared with those of several existing controllers^23^ under identical operating conditions. Tables 3 and 4 present the results for a 5% change in load power and a 5% change in wind power, respectively, highlighting the superior performance and advantages of the proposed controller in both steady-state and dynamic responses.

**Table 3:** Steady-state and dynamic characteristics for a 5% change in load power.

Controller type	Steady-state error	Settling time (sec)	Maximum overshoot	Rise time (sec)	Peak time (sec)
MOPSO-optimized µ-synthesis	7.0491e-9	10.31	1.5182e-7	2.9578e-5	1.76
MOGA-optimized µ-synthesis	2.7935e-8	10.95	5.8464e-7	1.1723e-4	1.81
Conventional µ-synthesis	9.0012e-7	18.822	2.5816e-6	0.00398	2.32
CHIO-optimized PID	2.0453e-5	30.2	7.3214e-5	0.0167	14.48
CHIO-optimized FOPID	7.3198e-6	28.1	4.8273e-6	0.00849	12.72

**Table 4:** Steady-state and dynamic characteristics for a 5% change in wind power.

Controller type	Steady-state error	Settling time (sec)	Maximum overshoot	Rise time (sec)	Peak time (sec)
MOPSO-optimized µ-synthesis	3.2e-9	12.7	1.0055e-7	1.3675e-5	1.51
MOGA-optimized µ-synthesis	2.8e-8	21.5	2.9599e-6	1.4752e-4	2.67
Conventional µ-synthesis	2.9e-7	23.8	8.6395e-6	0.0064	3.45
CHIO-optimized PID	4.3446e-5	32.6	8.4421e-5	0.04443	18.65
CHIO-optimized FOPID	4.2317e-6	30.1	5.6351e-6	0.00753	17.81

**Fig. 23:**
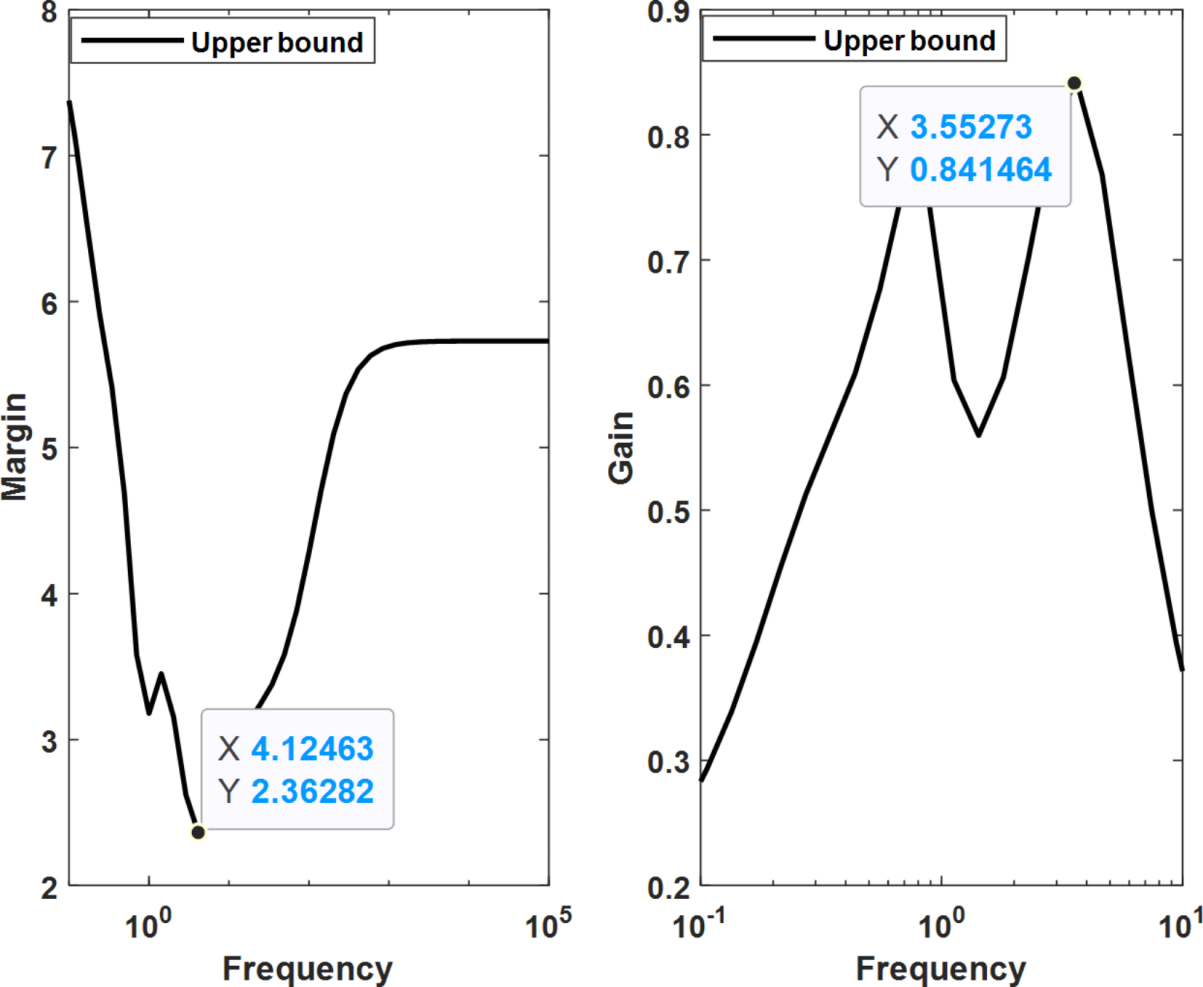
Robust characteristics for µ-synthesis controllers optimized by MOPSO.

**Fig. 24:**
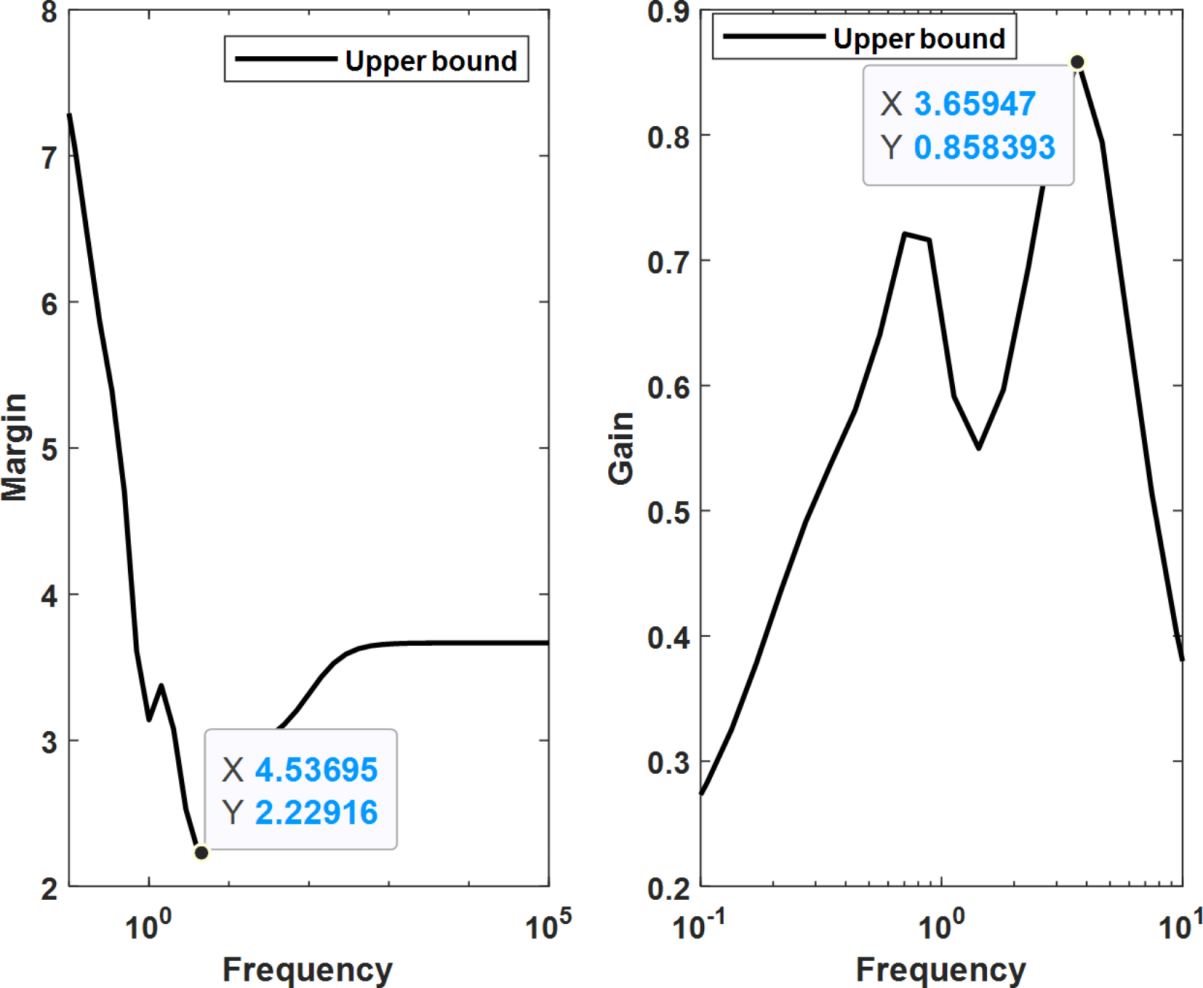
Robust characteristics for µ-synthesis controllers optimized by MOGA.

Robust stability and performance evaluations, conducted using the MATLAB toolbox, revealed that the optimal µ-synthesis MOPSO-optimized controllers can withstand up to 236% of the modeled uncertainty. This compares favorably to the 223% tolerance achieved by MOGA-optimized controllers and the 171% tolerance exhibited by conventional µ-synthesis controllers. Furthermore, the worst-case gain of the upper bound of µ was reduced to 0.84 for the MOPSO-optimized controller, compared to 0.85 for the MOGA-optimized controller and 0.98 for the conventional controller. These results highlight a substantial improvement in both robust performance and stability, showcasing the superior capability of the optimized controller to handle higher levels of system uncertainties and disturbances. This enhancement in performance underscores the effectiveness of the proposed optimization techniques in refining controller design for optimal microgrid frequency regulation. Figures 23 and 24 show the robust characteristics of µ-synthesis controllers optimized by MOPSO and MOGA, respectively.

The controller parameters are indicated in Table 5 for MOPSO and Table 6 for MOGA according to Eq. (18). Also, the weight parameters are indicated in Table 7 for MOPSO and Table 8 for MOGA according to Eq. (13).

**Table 5:** MOPSO controller parameters.

Subsystem	Controller parameters					
	$\:{\alpha\:}_{1}$	$\:{\alpha\:}_{2}$	$\:{\alpha\:}_{3}$	$\:{\beta\:}_{1}$	$\:{\beta\:}_{2}$	$\:{\beta\:}_{3}$
Diesel generator	-0.5851	22.51	73.38	1	71.28	4.714
Fuel cell	-0.8603	2	-3.76	1	71.28	4.714
Aqua-electrolyzer	-1.376	-1.299	0.1013	1	71.28	4.714
Ultra-capacitor	-1.796	-374.8	-41.8	1	71.28	4.714

**Table 6:** MOGA controller parameters.

Subsystem	Controller parameters					
	$\:{\alpha\:}_{1}$	$\:{\alpha\:}_{2}$	$\:{\alpha\:}_{3}$	$\:{\beta\:}_{1}$	$\:{\beta\:}_{2}$	$\:{\beta\:}_{3}$
Diesel generator	0.0247	40.16	107.1	1	89.14	7.414e-5
Fuel cell	-0.1904	5.459	-5.813	1	89.14	7.414e-5
Aqua-electrolyzer	0.1461	13.6	0.3684	1	89.14	7.414e-5
Ultra-capacitor	-4.451	-502.5	54.75	1	89.14	7.414e-5

**Table 7:** MOPSO weight parameters.

Subsystem	Weight parameters		
	$\:{K}_{w}$	$\:a$	$\:b$
Diesel generator	1	0.33383	0.82808
Fuel cell	0.249	0.32543	0.64461
Aqua-electrolyzer	0.55056	0.58509	0.59634
Ultra-capacitor	0.06388	0.96118	0.06997

**Table 8:** MOGA weight parameters.

Subsystem	Weight parameters		
	$\:{K}_{w}$	$\:a$	$\:b$
Diesel generator	1.09432	0.32048	0.81292
Fuel cell	0.23241	0.31891	0.65891
Aqua-electrolyzer	0.55102	0.60661	0.58531
Ultra-capacitor	0.0646	0.949	0.06226

The comparative analysis between MOPSO and MOGA shows that the MOPSO-optimized µ-synthesis controller consistently outperforms the MOGA-optimized and conventional controllers across multiple performance metrics, including uncertainty tolerance, frequency deviation control, and dynamic system response. These results validate the proposed optimization approach’s effectiveness and highlight its superiority in handling real-world uncertainties and disturbances, making it a more reliable choice for microgrid frequency regulation.

## Conclusions

This paper addresses the critical challenges of frequency regulation in isolated microgrids, complicated by system uncertainties and variable load demands, by developing an optimal µ-synthesis robust controller. A fixed-structure approach was employed to select performance and robustness weights tailored to the specific dynamic characteristics of each microgrid subsystem. Using MOGA and MOPSO optimization techniques with inequality constraints, the controller’s robust performance and stability were significantly enhanced. The comparative analysis demonstrated the proposed controller’s superiority over conventional µ-synthesis controllers, with the MOPSO-optimized design achieving a 236% increase in uncertainty tolerance compared to 171% for the conventional approach. The proposed controller also minimized frequency deviations and improved transient and steady-state performance. Nyquist stability analysis further validated its robust stability, confirming consistent performance across a wide range of uncertainties in renewable energy sources and confirming its ability to maintain consistent performance across a wide range of uncertainties in renewable energy sources.

The limitations of the µ-synthesis robust controller depend on the accuracy of the uncertainty representation model. If the model is inaccurate or the uncertainty bounds are not well-defined, the designed controller may fail to maintain robustness under various operating conditions. Future research will focus on implementing this controller in discrete-time and investigating its practical deployment in DSP systems for real-world microgrid applications. Furthermore, future work will explore the practical implications of this research for industry, emphasizing the commercial potential of the proposed solutions. This includes discussions on cost, scalability, and integration into existing systems—factors critical for assessing the commercial viability and long-term impact of the innovations presented.

## Data Availability

The datasets used and generated during the current study are available from the corresponding author upon reasonable request.

## Abbreviations

MOPSO: Multi-objective particle swarm optimization
MOGA: Multi-objective genetic algorithm
DER: Distributed energy resource
PV: Photovoltaics (PV)
ESS: Energy storage systems
MPC: Model predictive control
CD: Crowded distance
PID: Proportional-integral-derivative controller
FOPID: Fractional order proportional-integral-derivative controller
CHIO: Coronavirus herd immunity optimizer
DSP: Digital signal processors

## List of symbols

${\Delta P}_{L}$: Load power change
${\Delta P}_{D}$: Diesel generator power change
${\Delta P}_{F}$: Fuel cell power change
${\Delta P}_{WG}$: Wind generator power change
${\Delta P}_{A}$: Aqua-electrolyzer power change
${\Delta P}_{U}$: Ultra-capacitor power change
$${\alpha }_{P}$$: Uncertain parameter
$${P}_{\Delta }\left(S\right)$$: Normalized plant
$$P(S)$$: Nominal plant
${\mu }_{max}$: Maximum value of μ
${\mu }_{ub}$: Worst case gain for the upper bound of μ
${W}_{lb}$: Sensitivity function low pass filter
${W}_{hb}$: Complementary sensitivity high pass filter
${w}_{C}$: Minimum bandwidth frequency
${M}_{P}$: Maximum peak magnitude
$\varepsilon$: Maximum tracking error
${W}_{E}\left(s\right)$: Error weight transfer function
${W}_{GG}\left(s\right)$: Diesel generator weight transfer function
${W}_{FC}\left(s\right)$: Fuel cell weight transfer function
${W}_{AE}\left(s\right)$: Aqua-electrolyzer weight transfer function
${W}_{UC}\left(s\right)$: Ultra-capacitor weight transfer function
${C}_{\upmu }\left(S\right)$: μ-synthesis controller transfer function
